# Linear Combination Properties of Spectral Phasors Allow Thermodynamic Analysis of Reversible Denaturation of Multidomain and Membrane Proteins

**DOI:** 10.3390/ijms27209233

**Published:** 2026-10-17

**Authors:** Alvaro A. Recoulat Angelini, Leonel Malacrida, F. Luis González Flecha

**Affiliations:** 1 Laboratorio de Biofísica Molecular, Instituto de Química y Fisicoquímica Biológicas, Universidad de Buenos Aires—CONICET, Buenos Aires 1113, Argentina; 2 Universidad de Buenos Aires, Facultad de Farmacia y Bioquímica, Buenos Aires 1113, Argentina; 3 Unidad de Bioimagenología Avanzada, Institut Pasteur de Montevideo and Universidad de la República, Montevideo 11400, Uruguay; 4 Unidad Académica de Fisiopatología, Hospital de Clínicas, Facultad de Medicina, Universidad de la República, Montevideo 11600, Uruguay

**Keywords:** quantitative phasor analysis, protein folding and stability, membrane protein denaturation, unfolding intermediates

## Abstract

Denaturation of small globular proteins is often described as a reversible two-state transition between native and denatured conformations. Conversely, multidomain and membrane proteins frequently populate several partially folded intermediate states during denaturation. Traditional thermodynamic analysis of fluorescence intensity often fails to reliably identify these intermediate states. Alternatively, spectral phasors provide an effective model-free way to monitor these complex conformational changes by resolving overlapping fluorescence signals with small differences in the position or shape of the spectra. Nevertheless, converting phasor trajectories into quantitative thermodynamic information remains challenging when quantum yields do not remain constant during a given process, thus limiting their application to protein stability studies. In this work, we introduce a thermodynamic framework for quantifying protein stability from phasor trajectories. We validate this formalism using simulated spectral data generated from two- and three-state transitions, including changes in fluorescence quantum yields, successfully recovering the thermodynamic parameters used to generate the simulations. Additionally, we applied this approach to analyze published data on urea-induced unfolding of *E. coli* Outer Membrane Protein A. Phasor analysis identified a two-state transition, and the thermodynamic parameters agreed well with those obtained from total fluorescence intensity, supporting the validity of the proposed approach. Finally, applying this framework to the SDS-induced denaturation of *Archaeoglobus fulgidus* Cu(I)-transport ATPase enabled a complete thermodynamic characterization of the three transitions identified by phasor analysis of intrinsic fluorescence, which could not be resolved using fluorescence intensity alone. This formalism establishes fluorescence spectral phasors as a powerful quantitative approach for protein stability analysis.

## 1. Introduction

Protein stability is commonly defined as the thermodynamic resistance of the protein’s native folded conformation to disruption by environmental factors that lead to denatured states [1,2]. To explore this resistance, it is required: (a) the use of the so-called denaturing agents, (b) monitoring a signal that is sensitive to the conformational changes produced, and (c) finding the best mathematical model able to describe the behavior of the experimental data [1,3].

The simplest and most widely used model for describing protein denaturation is the so-called two-state model in which the process is represented as an elementary reversible chemical reaction. In this model, the native folded state of the protein (*N*) is the reactant and the denatured state of the protein (*D*) is the product:

*N* ⇌ *D* (1)

The Gibbs free energy change for the reversible transformation of the native state in the reference state (an ideal 1 M solution of the pure folded protein) to the denatured state, also in its reference state, can be expressed as:

(2) $${\Delta G}_{ND}^{\circ }=-R\cdot T\cdot \ln\frac{f_{D}}{f_{N}}$$

where f*_N_* and f*_D_* are the mole fractions of the native and denatured protein at equilibrium.

Determination of this free energy change requires altering both fractions along a fully reversible path. In this way, chemical denaturants preferentially interact with the expanded conformations of the polypeptide chains, stabilizing the unfolded state and/or destabilizing the native state, consequently decreasing the standard free energy difference between the folded and unfolded states [4]. As a first approximation, the dependence of the standard unfolding free energy on the denaturant concentration can be described by the first-order term of a Taylor expansion, leading to a linear relationship between these two quantities

(3) $$\Delta G_{ND}^{\circ }=\Delta G_{w,ND}^{\circ }+{\left(\frac{\partial {\Delta G}_{ND}^{\circ }}{\partial [\mathrm{denaturant}]}\right)}_{p,T}\cdot [\mathrm{denaturant}]={\Delta G}_{w,ND}^{\circ }-m_{ND}\cdot [\mathrm{denaturant}]$$

The term ΔG°*_w,ND_* represents the value of ΔG°*_ND_* in the absence of denaturant and is the established metric used to quantify protein stability.

The derivative of ΔG°*_ND_* with respect to the denaturant concentration is negative because increasing denaturant concentration shifts the equilibrium toward the denatured state. The absolute value of this derivative, known as the denaturant *m*-value, reflects the cooperativity of the denaturation process. Extensive experimental studies have shown that the *m*-values are strongly correlated with the increase in solvent-accessible surface area upon unfolding, indicating that proteins exposing larger surface areas to the solvent generally exhibit greater sensitivity to denaturants [5,6]. Consequently, the *m*-value is widely interpreted as a measure of the magnitude of the structural change associated with protein unfolding and of the differential interactions of the denaturant with the native and denatured states [7,8].

The two-state model described by Equations (1)–(3) has been extensively tested and validated for many small, single-domain globular proteins. Experimental support for the model comes from the observation of single unfolding transitions, agreement between equilibrium and kinetic measurements, and the correspondence between calorimetric and Van ’t Hoff thermodynamic parameters [9,10]. As a result, the two-state approximation has become a cornerstone of protein stability analysis and is widely used to interpret chemical- and thermal-denaturation experiments for small globular proteins [11,12].

Although the denaturation of large multidomain and membrane proteins can also be described by Equation (1), in such cases the equation should not be interpreted as describing an elementary chemical process. Instead, unfolding of these proteins typically proceeds through a series of partially folded intermediate species (corresponding to local minima in free energy landscapes) between the native (*N*) and denatured (*D*) conformations. Furthermore, multiple parallel unfolding pathways may connect the initial and final states, reflecting the structural complexity of these proteins. Despite this complexity, the signals and procedures used to monitor protein unfolding often fail to fully account for this scenario.

Suitable signals for quantitative unfolding studies must fulfill the condition of being, in a given mixture of chemical species, a linear combination of the specific signal of each species

(4) $$Signal_{mix}=\sum_{i}\;Signal_{i}\cdot f_{i}$$

This relation becomes simpler when (a) the intermediate states are poorly populated; (b) specific signals cannot be distinguished from either the one corresponding to the native state or the one corresponding to the denatured state; (c) parallel pathways occur concomitantly. In these scenarios, Equation (4) reduces to:

(5) $$Signal_{i}=Signal_{N}\cdot f_{N}+Signal_{D}\cdot f_{D}$$

and the system will be well described by a two-state model, independently of its complexity.

Fluorescence spectroscopy is a powerful and widely employed technique for quantitative investigation of protein folding [13,14]. This is due to its high sensitivity and, in a mixture of fluorescent species, the registered intensity satisfies Equation (4). Conversely, neither spectral shift nor spectral broadening can be used as signals for quantitative unfolding studies [15,16] because they do not meet the linear requirements expressed by Equation (4).

The conventional thermodynamic approach to protein unfolding studies involves monitoring a spectroscopic signal, most commonly the steady-state fluorescence of intrinsic aromatic residues, as a function of denaturant concentration. The resulting unfolding profile is then analyzed by fitting the equations derived for several unfolding models. The analysis usually starts with the two-state approximation and, when necessary, incorporates intermediate states until the simplest model capable of reproducing the experimental behavior is identified. This hierarchical approach enables obtaining information about the presence of stable partially folded intermediates along the unfolding pathway and calculating the corresponding thermodynamic parameters.

The phasor approach is a model-free tool, widely used in fluorescence imaging analysis [17,18,19]. It was introduced for lifetime fluorescence analysis by Enrico Gratton and David Jameson [20,21,22] on an original idea of Gregorio Weber [23] and extended to the study of fluorescence emission spectra by Farzad Fereidouni and coworkers [24]. In this framework, each spectrum is represented as a single point in a two-dimensional space denoted as the phasor plot, with the coordinates *G*_λ_ and *S*_λ_ obtained from the Fourier transformation of the spectral intensity distribution.

(6) $$G_{\lambda }=\frac{\sum_{\lambda }F(\lambda )\cdot \cos\frac{2\pi (\lambda -\lambda_{0})}{L}}{\sum_{\lambda }F(\lambda )}\;\mathrm{and}\;S_{\lambda }=\frac{\sum_{\lambda }F(\lambda )\cdot \sin\frac{2\pi (\lambda -\lambda_{0})}{L}}{\sum_{\lambda }F(\lambda )}$$

In these expressions, *F*(λ) represents the fluorescence intensity at wavelength λ, λ_0_ is the initial wavelength of the spectrum, and *L* corresponds to the spectral range (*L* = λ_final_ − λ_0_).

A phasor plot was built by representing the imaginary component of the Fourier transform *S*_λ_ as a function of the real component *G*_λ_.

The phasor reflects global properties of the spectrum, such as its shape and spectral position, allowing complex datasets to be visualized in a reduced-dimensional space. Because this representation is generated directly from the experimental data and does not require fitting procedures or predefined mechanistic models, this type of analysis is considered a model-independent method [25].

Spectral phasors have proven particularly useful for investigating protein conformational changes, where multiple states may coexist and produce overlapping fluorescence signals. By monitoring the evolution of phasor coordinates under different conditions, folding transitions and intermediate conformations can be identified and characterized in a straightforward manner. Thus, phasor analysis serves as a valuable complement to conventional thermodynamic approaches, providing an intuitive framework for exploring complex folding landscapes [26,27]. In a recent work [28], we used the combination of traditional thermodynamic analysis with the model-free phasor analysis to explore the reversible denaturation induced by the detergent SDS of a helical membrane protein, the Cu(I) transport ATPase from *Archaeoglobus fulgidus* (*Af*CopA). Despite its multidomain architecture, *Af*CopA thermal- and guanidinium chloride-induced denaturation were characterized as two-state processes [29,30]. To further explore its conformational stability and unfolding mechanism, we monitored SDS-induced structural changes by tryptophan fluorescence spectroscopy. Conventional thermodynamic analysis of the resulting fluorescence data supported an unfolding pathway involving a single intermediate state. In contrast, spectral phasor analysis of the same dataset revealed a more complex conformational landscape with two distinct intermediates. These findings demonstrated that phasor analysis can uncover structural information that remains inaccessible to traditional thermodynamic approaches [28].

In addition to its power as a qualitative, model-free tool to analyze fluorescence data, the linear properties of phasors derived from its vectorial nature open the possibility of performing quantitative analysis [31]. For instance, in their fluorescence lifetime phasor analysis of the decamer-dimer equilibrium of human peroxiredoxin 1, Villar et al. [32] demonstrated that the fluorescence quantum yield remains invariant during the decamer formation/dissociation. Under this condition, the observed phasor trajectory was attributed to shifts in population distributions among the oligomeric species, allowing the authors to calculate the equilibrium constant for this process.

In this work, we analyze the mathematical properties of phasors that enable their use in quantitative analysis of reversible protein denaturation processes, taking into account that changes in fluorescence quantum yields are a common feature in these processes. The proposed formalism is then validated using simulated and published data and used to analyze the SDS-induced denaturation of *Af*CopA.

## 2. Results and Discussion

### 2.1. Theory and Simulations

As was mentioned in the introduction, a phasor plot is the graphical representation of phasors in which each fluorescence spectrum is represented by a single point with coordinates *G*_λ_ and *S*_λ_. When studying a process in which the fluorescence spectrum changes its spectral position or shape, each spectrum taken along the reaction coordinate will be represented by a different point in the phasor plot, thus defining a trajectory.

**Figure 1:**
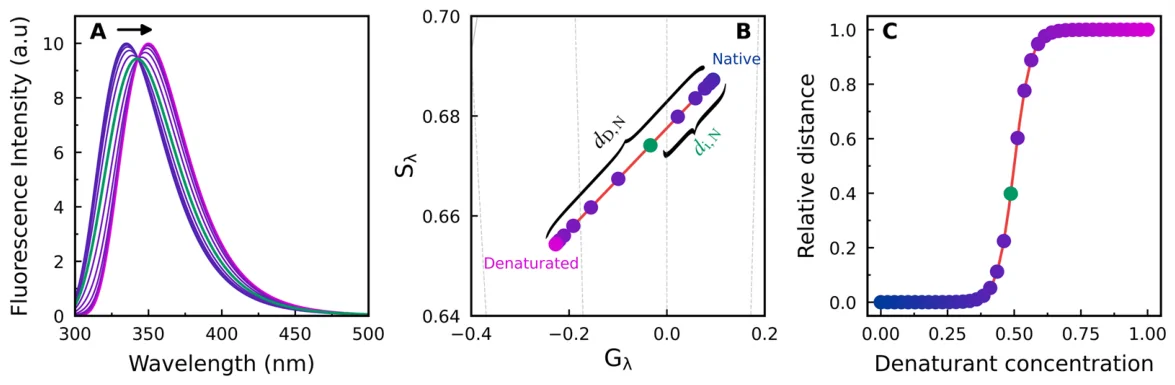
Simulation of a two-state denaturation process exhibiting a fluorescence red shift without changes in the quantum yield. (**A**) Fluorescence spectra of the native (blue) and denatured (purple) states were simulated using a log-normal function [33]. Both spectra were simulated with the same spectral width at half-maximum but with shifted emission maxima (native at 335 nm and denatured at 350 nm). A denaturation process was modeled using Equations (2) and (3) with ΔG°*_w,ND_* = 40 kJ/mol and *m_ND_* = 80 kJ/mol, yielding the fractions of native (f_N_) and denatured (f_D_) species corresponding to different denaturant concentrations. With this data, the spectra along the denaturation process were constructed as a linear combination of the native and denatured spectra (Equation (17)). The arrow indicates increasing denaturant concentration. (**B**) Phasor plot of the simulated spectra. Phasor components of each spectrum shown in panel A were calculated using Equation (6). As the transition progresses, the phasor point of a given spectrum (e.g., the green point “i”, corresponding to the green spectrum in panel A) falls along a straight line connecting the phasors of the native and denatured spectra. (**C**) The relative distances in the phasor plot (*d*_i,N_/*d*_D,N_) were calculated using Equation (18) and plotted as a function of the denaturant concentration (expressed as relative to the maximal concentration). Equation (16), corresponding to a two-state model, was fitted to these data considering equal quantum yields. The best-fit parameter values were ΔG°*_w,ND_* = 40 ± 10^−6^ kJ/mol and *m_NU_* = 80 ± 10^−6^ kJ/mol, thus recovering the original values used in the simulation.

Figure 1 shows a typical trajectory in the phasor space for a two-state protein denaturation process. Points in this figure represents the phasor transformation corresponding to the fluorescence spectra of the protein before and after the addition of increasing concentrations of denaturant. These phasors are vectors in the complex space, and the component *G*_i_ of the vector for a given addition of denaturant can be expressed as a linear combination of the real components of the phasors corresponding to the native and denatured states (*G_N_* and *G_D_*). Each component is pondered by the fraction of photons arriving at the detector from protein molecules in the native or denatured state (pf*_N,i_* and pf*_D,i_*) corresponding to a given addition of denaturant.

(7) $$G_{i}=G_{N}\cdot {\mathrm{pf}}_{N,i}+G_{D}\cdot {\mathrm{pf}}_{D,i}=G_{N}\cdot \left(1-{\mathrm{pf}}_{D,i}\right)+G_{D}\cdot {\mathrm{pf}}_{D,i}=G_{N}+\cdot \left(G_{D}-G_{N}\right)\cdot {\mathrm{pf}}_{D,i}$$

Similarly, the imaginary component of the phasor (*S*_i_) will be:

(8) $$S_{i}=S_{N}+\left(S_{D}-S_{N}\right)\cdot {\mathrm{pf}}_{D,i}$$

It is clear the similarity of Equations (7) and (8) with Equation (5), thus validating that the phasor components fulfill the linear dependence required for signals to be used in quantitative analysis.

On the other hand, the algebraic properties of vectors allow us to calculate the distance between the phasors corresponding to the native and the denatured states, which is given by:

(9) $$d_{D,N}=\sqrt{{\left(G_{D}-G_{N}\right)}^{2}+{\left(S_{D}-S_{N}\right)}^{2}}$$

and, in a similar way, the distance between each intermediate stage (composed of a mixture of native and denatured molecules) and the native state can be expressed as:

(10) $$d_{i,N}=\sqrt{{\left(G_{i}-G_{N}\right)}^{2}+{\left(S_{i}-S_{N}\right)}^{2}}$$

Replacing Equations (7) and (8) in Equation (10)

(11) $$d_{i,N}=\sqrt{{\left([G_{N}+(G_{D}-G_{N})\cdot {\mathrm{pf}}_{D,i}]-G_{N}\right)}^{2}+{\left([S_{N}+(S_{D}-S_{N})\cdot {\mathrm{pf}}_{D,i}]-S_{N}\right)}^{2}}$$

In each quadratic term within the squared root, the phasor component corresponding to the native state appears as both an additive and a subtractive term, and therefore cancels out. Furthermore, the resulting terms have a common factor of pf*_D,i_*^2^, thus:

(12) $$d_{i,N}=\sqrt{\left({\left(G_{D}-G_{N}\right)}^{2}+{\left(S_{D}-S_{N}\right)}^{2}\right)\cdot {\mathrm{pf}}_{D,i}^{2}}$$

Defining the relative distance (*rd*) as the quotient between the distance from the phasor corresponding to each addition of denaturant and that of the native state and the full distance between the phasors corresponding to the native and the fully denatured state, we obtain:

(13) $$rd=\frac{d_{i,N}}{d_{D,N}}=\frac{\sqrt{\left({\left(G_{D}-G_{N}\right)}^{2}+{\left(S_{D}-S_{N}\right)}^{2}\right)\cdot {\mathrm{pf}}_{D,i}^{2}}}{\sqrt{{\left(G_{D}-G_{N}\right)}^{2}+{\left(S_{D}-S_{N}\right)}^{2}}}=\sqrt{{\mathrm{pf}}_{D,i}^{2}}={\mathrm{pf}}_{D,i}$$

This means that the relative distance defined by Equation (13) equals the fraction of photons arriving at the detector emitted from molecules in the denatured state.

On the other hand, as shown in Appendix A, the fraction of molecules in the denatured state is related to the corresponding fraction of photons (which is equal to *rd*) as:

(14) $$f_{D}\;=\;\frac{rd\cdot \phi_{N}}{\phi_{D}+rd\cdot (\phi_{N}-\phi_{D})}$$

where *ϕ_N_* and *ϕ_D_* are the fluorescence quantum yields of the fluorophores in the native and denatured protein.

It is easy to see that if the quantum yields of both species are equal, both fractions are equal to each other and equal to the relative distance in the phasor space.

Replacing now Equation (14) in Equation (2), and considering that for a two-state process f*_N_* + f*_D_* = 1, we obtain an expression of the standard free energy change in the reference states as a function of the relative distance in the phasor space

(15) $$\Delta G_{ND}^{\circ }=-R\cdot T\cdot \ln\frac{rd\cdot \phi_{N}}{\left(1-rd\right)\cdot \phi_{D}}$$

Resolving this equation for *rd*

(16) $$rd=\frac{\phi_{D}}{\phi_{D}+\phi_{N}\cdot e^{\frac{\Delta G_{w,\mathrm{ND}}^{\circ }-m_{\mathrm{ND}}\cdot [\mathrm{denaturant}]}{R\cdot T}}}$$

To evaluate this formalism, we performed a series of computational experiments in which pseudoexperimental datasets were generated from predefined thermodynamic parameters describing protein folding under a given model. The resulting datasets were then analyzed using the proposed formalism to assess its ability to correctly recover both the underlying folding mechanism and the corresponding parameter values.

First, we simulated a two-state unfolding process in which the native and denatured states differed exclusively in their spectral position, while the spectral shape and quantum yield remained unchanged.

The denaturation process was modeled using Equations (2) and (3) with selected values of ΔG°*_w,ND_* and *m_ND_*, yielding the fractions of native (f*_N_*) and denatured (f*_D_*) species corresponding to different denaturant concentrations. With this data, the spectra along the denaturation process were constructed as a linear combination of the native and denatured spectra

(17) $$F\left(\lambda \right)=f_{N}\cdot F{\left(\lambda \right)}_{N}+f_{D}\cdot F{\left(\lambda \right)}_{D}$$

Figure 1A shows the fluorescence emission spectra generated at increasing denaturant concentrations. The corresponding phasors are displayed in Figure 1B. As expected for a two-state transition, all phasors lie on the straight line connecting the phasors of the native and fully denatured states. This linear trajectory reflects the fact that each spectrum can be represented as a weighted combination of only two fluorescent species and demonstrates that the phasor representation faithfully captures the two-state nature of the simulated unfolding process. Quantitative analysis was performed by calculating the relative position of each phasor along the transition coordinate for each denaturant concentration as:

(18) $$rd=\frac{\sqrt{{\left(G_{i}-G_{N}\right)}^{2}+{\left(S_{i}-S_{N}\right)}^{2}}}{\sqrt{{\left(G_{D}-G_{N}\right)}^{2}+{\left(S_{D}-S_{N}\right)}^{2}}}$$

Then, Equation (16) was fitted to the resulting set of data. This procedure successfully recovered both the standard unfolding free energy in the absence of denaturant and its derivative with respect to denaturant concentration. These values were consistent with those obtained by fitting a conventional two-state model Equation (21) to the fluorescence intensity at 350 nm (Figure S1), demonstrating agreement between both methodologies and confirming the ability of the proposed formalism to recover the thermodynamic parameters used to generate the simulated data.

A similar result is obtained when the native and denatured states differ only in spectral shape (i.e., in the distribution of the emitted energies), while their total fluorescence intensity and spectral maximum remain unchanged. In the simulation shown in Figure S2, the denatured state exhibits a broader emission spectrum than the native state, and the corresponding phasors again follow a linear trajectory characteristic of a two-state transition, and the thermodynamic parameter values are accurately recovered.

Taken together, these results clearly demonstrate that the phasor representation provides a straightforward means of establishing the two-state nature of a process in which protein denaturation is reflected solely by spectral shifts or changes in spectral shape, without requiring the introduction of models of increasing complexity associated with protein denaturation. Moreover, the linear properties of the phasor transformation allow accurate determination of thermodynamic denaturation parameters, a capability that cannot be achieved through the analysis of changes in spectral maxima or spectral width at half maximum as a function of denaturant concentration (Figures S1C and S2C).

An important exception arises when the native and denatured states differ exclusively in their total fluorescence intensity. In this case, because phasor coordinates are normalized quantities, both states are represented by the same point in phasor space, and all intermediate spectra generated along the denaturation transition collapse onto that same point. Consequently, the phasor approach cannot resolve a denaturation process with these characteristics. However, such transitions can still be readily characterized by conventional thermodynamic analysis of fluorescence intensity changes. This limitation highlights the complementary nature of the phasor and intensity-based approaches, as each is sensitive to different spectroscopic manifestations of protein unfolding.

Having demonstrated the ability of the proposed formalism of quantitatively analyzing trajectories in the phasor space, we next consider a more general scenario that more closely reflects the conditions encountered in a real protein denaturation experiment: the case in which the fluorescence spectrum of the denatured state differs from that of the native state in fluorescence quantum yield, spectral position, and full width at half maximum (Figure S3). In this more general case, the two-state nature of the unfolding transition and the thermodynamic parameter values characterizing this transition are still accurately recovered. Importantly, fitting Equation (16) with *ϕ_N_ =* *ϕ_D_* leads to an incorrect estimation of the Gibbs free energy change, despite the excellent quality of the fit. This result highlights the importance of explicitly accounting for differences in quantum yield when extracting thermodynamic information from phasor trajectories.

**Figure 2:**
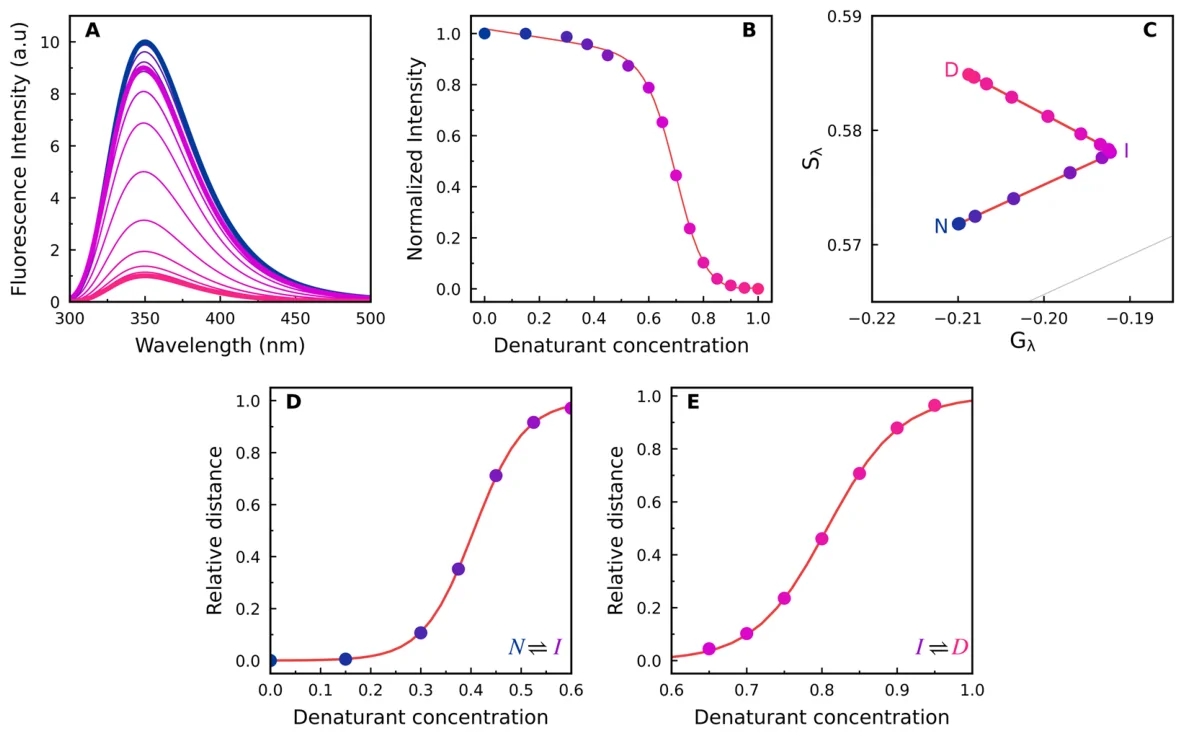
Simulation of a three-state denaturation process with an intermediate state hidden from traditional thermodynamic analysis. (**A**) Fluorescence spectra of the native (blue), intermediate (purple), and denatured (red) states were simulated using a log-normal function [33]. The intermediate-state spectrum was simulated with the same spectral width at half maximum as the native state, but *ϕ_I_*/*ϕ_N_* = 0.9 and a 1 nm blue shift in the emission maximum. The denatured-state spectrum was simulated with a broader spectral width at half maximum, *ϕ_D_*/*ϕ_I_* = 0.11, and the same emission maximum as the intermediate state. A denaturation process was modeled using Equations (2) and (3) for each transition with ΔG°*_w,NI_* = 20 kJ/mol, *m_NI_* = 50 kJ/mol, ΔG°*_w,ID_* = 35 kJ/mol and *m_ID_* = 50 kJ/mol yielding the fractions of native (f*_N_*), intermediate (f*_I_*) and denatured (f*_D_*) species corresponding to different denaturant concentrations. With this data, the spectra along the denaturation process were constructed as a linear combination of the native, intermediate and denatured spectra. (**B**) The total fluorescence intensity was calculated, normalized using Equation (19), and plotted as a function of denaturant concentration (expressed as relative to the maximal concentration). Equation (21) was fitted to these data. The best-fit parameter values were ΔG°*_w,ND_* = 32 ± 2 kJ/mol and *m_ND_* = 46 ± 3 kJ/mol, including a slope of the native baseline versus denaturant concentration (*m_N_*) of −0.17 ± 0.04. (**C**) Phasor components of each spectrum shown in panel A were calculated using Equation (6). The relative distances in the phasor plot between the native and the intermediate state (*d_i,N_*/*d_I,N_*) and the intermediate and the denatured state (*d_i,I_*/*d_D,I_*) were calculated using Equation (18) and plotted as a function of the denaturant concentration in panels (**D**) and (**E**), respectively. Equation (16) was fitted to these data (red line). The best-fit parameter values were ΔG°*_w,NI_* = 20.0 ± 0.01 kJ/mol, *m_NI_* = 50.0 ± 0.04 kJ/mol, ΔG°*_w,ID_* = 35.0 ± 0.2 kJ/mol and *m_ID_* = 50 ± 0.3 kJ/mol, thus recovering the original values used in the simulation.

The next step in complexity is a three-state denaturation process (Figure 2). As mentioned in the Introduction, the existence of unfolding intermediates can be overlooked by traditional thermodynamic analyses that rely exclusively on fluorescence intensity measurements. To illustrate this scenario, the emission spectrum of the intermediate state was simulated with a fluorescence intensity 10% lower than that of the native state, a 1 nm blue shift in the emission maximum, and an unchanged spectral shape. It is worth noting that spectral blue-shift upon protein denaturation is a common feature in SDS-induced denaturation of proteins (e.g., [34,35]). When only fluorescence intensity data are considered, the unfolding process is well-described by an apparent two-state model (Figure 2B). However, the thermodynamic parameters obtained from this analysis do not correspond to the values used in the simulation, nor to their sum or average, indicating that hidden intermediate states lead to an incorrect estimation of the stability parameters as previously suggested for multidomain proteins [36]. It is worth noting that, in this error-free spectral simulation, a three-state model can be fitted to the total intensity data and can effectively recover the thermodynamic parameters used to generate the simulation. However, introducing only 1% random error results in substantially different parameter estimates. In contrast, phasor analysis clearly reveals the presence of the intermediate state, and two linear trajectories were identified (Figure 2C). Furthermore, the formalism proposed in this work accurately recovers the standard unfolding free energies and their derivatives with respect to denaturant concentration for both unfolding steps.

Once we have verified that the proposed formalism is effective in recovering the information underlying the simulated data, we proceed to use it for the analysis of experimental data.

### 2.2. Urea-Induced Denaturation of a Beta Barrel Outer Membrane Protein

Outer membrane proteins (OMPs) comprise a structurally and functionally diverse family of β-barrel proteins involved in processes such as nutrient uptake, molecular transport, signal transduction, and outer membrane biogenesis and maintenance in Gram-negative bacteria [37]. Although OMPs represent only a small fraction of membrane proteins [38], they play essential roles in the physiology and viability of these bacteria [39]. Their unique architecture and the complexity of their folding and membrane insertion pathways have made OMPs a major model system for investigating membrane protein folding, stability, and assembly mechanisms [40,41].

OmpA of *Escherichia coli* (*Ec*OmpA) is one of the most extensively studied and highly abundant proteins anchored to the outer membrane [42]. Structurally, it is composed of a transmembrane domain, containing eight-stranded antiparallel β-strands arranged in an all-next-neighbor topology [43], as shown in Figure S4A, and a periplasmic domain with a globular structure [44]. OmpA fulfills several structural and physiological roles, including maintaining cell morphology and integrity, contributing to biofilm development, functioning as an adhesin, invasin, and evasin, acting as a receptor for colicins and bacteriophages, and facilitating F-dependent conjugation [45].

*Ec*OmpA has five tryptophan residues, all of them located in transmembrane β-strands, thus reporting on the tertiary structure of the transmembrane domain. Urea-induced equilibrium unfolding of OmpA—monitored through the intrinsic fluorescence—was reported to follow a two-state process ([46] and references therein). To investigate how the position of lipid-facing tryptophan residues within the membrane bilayer affects *Ec*OmpA stability, Tamm and collaborators [47] designed a quadruple alanine mutant in the first transmembrane β-strand (W_0_-*Ec*OmpA). This mutant served as a template for the introduction of single lipid-faced tryptophan residues at different positions across the bilayer. Urea-induced unfolding of these mutants led to a decrease in intrinsic fluorescence, and the reversibility of this process enabled a thermodynamic analysis in which the unfolding of all mutants could be described by a two-state model. The authors conclude that lipid-facing tryptophan residues contribute to *Ec*OmpA stability, with residues near the bilayer center being slightly more stabilizing than those at the membrane interface when water was used as the reference state.

**Figure 3:**
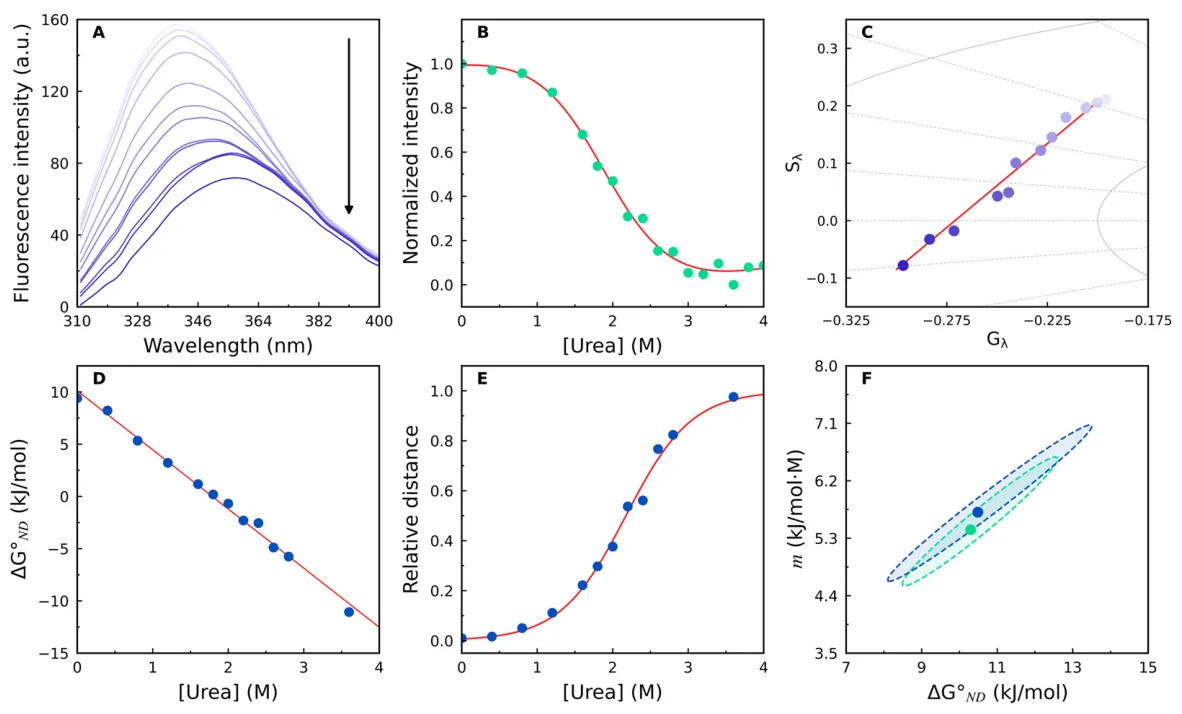
Urea-induced denaturation of *E. coli* outer membrane protein A (W_0_-*Ec*OmpA). (**A**) Intrinsic fluorescence spectra of *Ec*OmpA at different urea concentrations (blue lines with increasing color saturation) were digitized from a previously published study [47]. The arrow indicates increasing urea concentration. (**B**) The total fluorescence emission of each spectrum was calculated, normalized using Equation (19), and plotted against urea concentration. A two-state model (Equation (21)) was fitted to these data, and the best-fit parameters are reported in Table 1. (**C**) Phasor plot of the spectra shown in panel (**A**). Phasor components of each spectrum were calculated using Equation (6) and represented in a phasor plot. (**D**) ΔG°*_ND_* values were calculated for each urea concentration using Equation (15) and plotted against urea concentration. The red line shows the linear regression of the dataset, yielding a slope of −5.7 ± 0.3 kJ·mol^−1^·M^−1^ and an intercept of 10.5 ± 0.7 kJ·mol^−1^. (**E**) The relative distances in the phasor plot were calculated using Equation (18) and plotted as a function of the denaturant concentration. Equation (16) was fitted to these data (red line), and the best-fit parameters are reported in Table 1. (**F**) The 99% confidence regions (dotted lines) for the best-fit parameters values ΔG°*_w,ND_* and *m_ND_* (points) were obtained from the fitting to the total intensity data (green) and to the relative distance in the phasor plot (blue).

To extend our analysis to previously published data, we digitized the intrinsic fluorescence spectra of one of the mutants recorded by the authors at different urea concentrations (Figure 3A).

Figure 3B shows the typical pattern of a two-state unfolding curve. On the other hand, the phasor transformation of the spectra reveals a linear trend (Figure 3C), also consistent with a two-state transition. We estimate the quantum yields of the native state (spectra of *Ec*OmpA in the absence of urea) and the unfolded state (spectra of *Ec*OmpA at 4 M of urea). In addition, the calculated Gibbs free energy of unfolding (ΔG°*_w,ND_*) shows a linear dependence on urea concentration (Figure 3D), verifying the linear approximation described by Equation (3). Taking this into account, we use the procedure proposed in this work to describe the dependence of the relative distance in the phasor plot on urea concentration (Figure 3E).

The best-fit parameter values obtained by fitting the two-state model to either the total fluorescence data (Equation (21)) or the relative distance in the phasor plot (Equation (16)) are similar and are in agreement with the values reported in the original publication [47]. To further evaluate the statistical agreement between both fits, 99% confidence regions for the best-fit values of ΔG°*_w,ND_* and *m_ND_* (Figure 3F) were generated. The observed overlap indicates that both signal-processing approaches evaluate the same population of model parameters.

To highlight the importance of the formalism proposed in this work, we probe the fitting of Equation (16) without considering the differences in quantum yields between both states (Figure S5). It can be observed that, although the model well describes the dependence of the relative distance on the urea concentration, the ΔG°*_w,ND_* value obtained using this procedure (12.4 ± 0.7 kJ·mol^−1^) strongly differ from that reported in Table 1 (see also the non-overlapped 99% confidence regions in Figure S5C).

### 2.3. SDS-Induced Denaturation of a Helical Membrane Protein

Helical membrane proteins constitute the largest class of integral membrane proteins and are responsible for a wide variety of essential cellular functions, including transport across biological membranes, signal transduction, energy conversion, and cell communication [45,48]. Approximately 20–30% of the proteins encoded in most genomes are membrane proteins, with the vast majority belonging to the α-helical class [49]. Owing to their central physiological roles, helical membrane proteins comprise many of the most therapeutically relevant drug targets, with drugs targeting G protein-coupled receptors alone accounting for approximately one-third of all drugs approved by the US Food and Drug Administration [50].

*Af*CopA is a Cu(I)-transport P-type ATPase from the hyperthermophilic archaea *Archaeoglobus fulgidus* that has been the subject of extensive structural and functional studies (e.g., [51,52,53,54,55]). Structurally, it comprises a transmembrane domain containing eight transmembrane α-helices arranged into a compact helical bundle, as well as four cytoplasmic domains (Figure S4B), in agreement with the overall architecture described for other structurally characterized P_IB_-type ATPases [56,57]. These include the phosphorylation and nucleotide-binding domains (P- and N-domains), both constituting the catalytic unit, the actuator domain (A-domain), and two metal-binding domains located at the N- and C-termini [55]. Functionally, it is involved in maintaining cellular copper homeostasis by exporting Cu(I) across biological membranes in a process coupled to ATP hydrolysis [58].

*Af*CopA has three tryptophan residues, two of them located in transmembrane helices and the third in the catalytic unit, thus reporting on the tertiary structure of both regions. As previously mentioned, SDS can reversibly denature *Af*CopA [28]. The structural changes induced by the detergent were monitored by intrinsic tryptophan fluorescence spectroscopy, which showed a progressive loss of fluorescence intensity upon SDS addition. When analyzed as a function of the SDS concentration in the micellar phase, estimated through an iterative procedure [59], the fluorescence intensity data were well described by a three-state model, containing a single intermediate state. However, spectral phasor analysis of the same dataset revealed a more complex conformational landscape, identifying two distinct intermediates connected by linear trajectories in phasor space.

**Figure 4:**
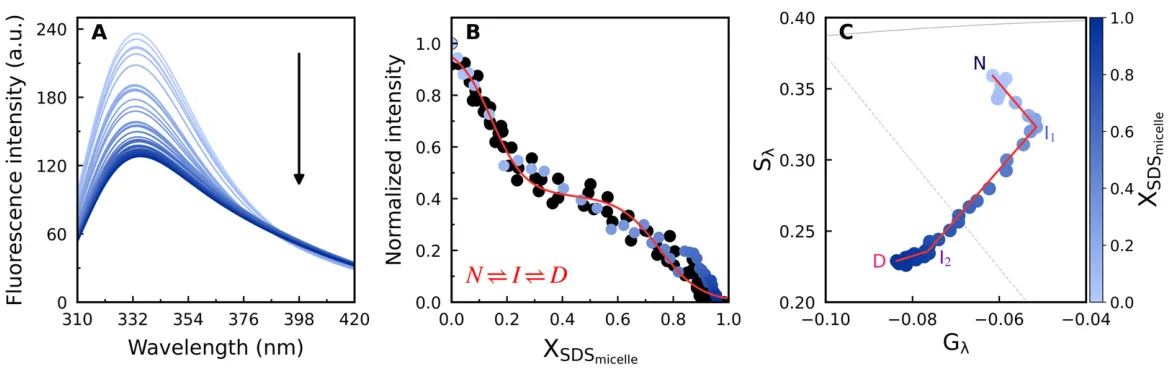
SDS-induced denaturation of *Archaeoglobus fulgidus* Cu(I) transport ATPase. (**A**) *Af*CopA (2 μM) was incubated for 5 min at 25 °C in the medium described in Materials and Methods with increasing concentrations of SDS (indicated by increasing blue color saturation) in two independent titrations. Intrinsic fluorescence spectra were recorded between 310 and 420 nm after excitation at 295 nm. The arrow indicates increasing SDS concentration. (**B**) The total fluorescence intensity was calculated, normalized using Equation (19), and plotted against the mole fraction of SDS in the micelle (see Materials and Methods for details of this calculation). Black dots represent the experimental data from a previous study [28], and the red line corresponds to a three-state model (Equation (22)) fitted to this data. The best-fit parameter values were ΔG°_w,NI_ = 5.5 ± 0.5 kJ·mol^−1^, m_NI_ = 40 ± 3 kJ·mol^−1^, ΔG°_w,ID_ = 25 ± 2 kJ·mol^−1^, m_ID_ = 33 ± 3 kJ·mol^−1^. (**C**) Phasor plot of the spectra shown in panel A. Phasor components of each spectrum were calculated using Equation (6) and represented in a phasor plot. The red lines indicate three linear transitions, consistent with the presence of four species during the denaturation process: Native (N), intermediary 1 (I_1_), intermediary 2 (I_2_), and denatured states (D).

To further explore this scenario, Trp fluorescence spectra of *Af*CopA were registered after incubation with different SDS concentrations (Figure 4A).

It can be observed that both the total fluorescence intensity pattern (Figure 4B) and the phasor trajectory (Figure 4C) cannot be explained with a two-state denaturation model. In the case of the dependence of the total fluorescence intensity on the SDS mole fraction, the best fit was achieved with a three-states model, but a bias on fitting is observed for high SDS mole fractions. This bias is avoided if a second intermediate was included in the model, but this results in overparameterization and thus cannot be taken into account in this thermodynamic analysis.

Application of the formalism described in this work requires estimating the fluorescence quantum yield of each species identified along the denaturing pathway. The three transitions followed well-defined linear trajectories in phasor space; thus, the vertices could be directly assigned to intermediate species (Figure S6). The fluorescence quantum yields associated with each identified state were determined as described in Materials and Methods, and the results are shown in Table S1. On the other hand, reversibility of the denaturation process was verified by the recovery of the spectral characteristic after removal of SDS by dialysis and the full recovery of the ATPase activity.

**Figure 5:**
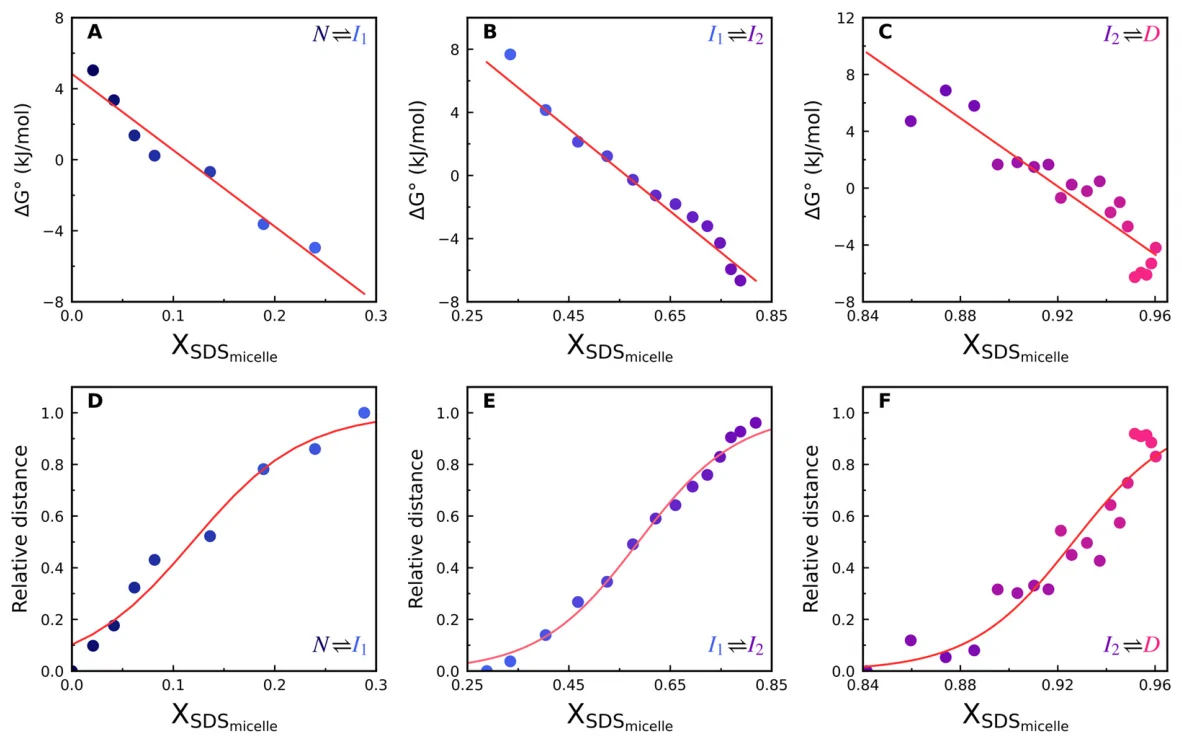
Thermodynamic analysis of SDS-induced denaturation of *Af*CopA based on the phasor plot. For each transition identified in the phasor plot (Figure 4C), the relative distances were calculated using Equation (18), and the Gibbs free energy of each transition was determined from the quantum yield–weighted relative distance given by Equation (15) and plotted accordingly (panels (**A**–**C**)). Linear fits to the data (red lines) yield the following relationships: −42 ± 4 kJ·mol^−1^·X_SDSmic_ + 4.8 ± 0.6 kJ·mol^−1^ (panel (**A**)), −26 ± 2 kJ·mol^−1^·X_SDSmic_ + 14 ± 1 kJ·mol^−1^ (panel (**B**)), and −120 ± 12 kJ·mol^−1^·X_SDSmic_ + 110 ± 11 kJ·mol^−1^ (panel (**C**)). The relative distances in each transition were plotted as a function of the mole fraction of SDS in the micelle (panels (**D**–**F**)). Each transition was analyzed by fitting Equation (16), and the corresponding best-fit parameter values are summarized in Table 2.

With this information, and considering that the molar absorption coefficient of *Af*CopA is not affected by the addition of SDS (Figure S7), we calculated the fractions of each species from the relative distances on the phasor data and the Gibbs free energy of each process for each SDS mole fraction (Figure 5A–C). The resulting plots showed a linear correlation between the standard Gibbs free energy and the denaturant concentration, thus validating the assumption given by Equation (3). It is worth noting that, while the validity of this relationship is well established for soluble proteins, its applicability for measuring the stability of membrane proteins has to be probed in each case [60].

Taking this into account, we fit our formalism to the dependence of the relative distance in the phasor plot on the mole fraction of SDS corresponding to each transition (Figure 5D–F).

**Table 2:** Best-fit parameter values for SDS-induced denaturation of *Af*CopA.

Fluorescence Total Intensity			Spectral Phasor Analysis		
Transition	ΔG°_w_ (kJ·mol^−1^)	*m* (kJ·mol^−1^)	Transition	ΔG°_w_ (kJ·mol^−1^)	*m* (kJ·mol^−1^)
*N* ⇋ *I*	5.5 ± 0.5	40 ± 3	*N* ⇋ *I*_1_	4.9 ± 0.6	45 ± 6
*I* ⇋ *D*	25 ± 2	33 ± 3	*I*_1_ ⇋ *I*_2_	14.5 ± 0.8	25 ± 1
			*I*_2_ ⇋ *D*	109 ± 14	117 ± 15

The standard free energy change associated with the first stage of the denaturation process, calculated using both procedures, shows no significant differences (Figure S8). In contrast, the ΔG° value assigned to the second stage from the fluorescence intensity data using the conventional thermodynamic analysis does not correspond to any of the values derived from the last two linear regions of the phasor plot. This behavior is consistent with that observed for the simulated three-state process (Figure 2), in which the presence of an intermediate, not resolved by the intensity-based analysis, leads to an inaccurate estimation of the thermodynamic parameters. These results highlight the power of the phasor analysis to resolve additional intermediates that are masked in the conventional thermodynamic analysis, but at the same time point out a note of caution about the interpretation of thermodynamic stability data in multidomain proteins that show a complex unfolding landscape.

## 3. Materials and Methods

### 3.1. Chemicals

Ultrapure sodium dodecyl sulfate (SDS) was purchased from MP Biomedicals (Santa Ana, CA, USA). Polyoxyethylene (10) lauryl ether (C_12_E_10_) and soybean phospholipids (asolectin) were obtained from Sigma-Aldrich (St. Louis, MO, USA). All other chemicals were of analytical grade and were purchased from Sigma-Aldrich (St. Louis, MO, USA). Solutions were prepared with glass-distilled water, and pH values were adjusted immediately before each experiment.

### 3.2. Protein Expression and Purification

*Af*CopA cDNA was cloned into the pBAD-TOPO/His expression vector, and recombinant protein expression in *Escherichia coli* Top10 cells was performed as previously reported [61,62]. Membranes obtained from *Af*CopA-expressing cells were solubilized in 16 mM C_12_E_10_. *Af*CopA was purified by Ni^2+^-affinity chromatography, and the immobilized protein was thoroughly washed to eliminate other membrane proteins. Following elution, an additional purification step was carried out by size-exclusion chromatography using a Superdex 200 column. Protein integrity and purity were evaluated by Tris-tricine SDS-PAGE [63], and protein concentration was determined by light absorbance using the molar absorption coefficients at 280 nm calculated from their sequence [64] (39,700 M^−1^ cm^−1^), after correcting the absorption spectra for light scattering [65]. Purified *Af*CopA was stored at −70 °C in a buffer containing 25 mM Tris-HCl (pH 7.0), 50 mM NaCl, 0.16 mM C_12_E_10_, and 100 mM sucrose.

### 3.3. SDS-Induced Denaturation of AfCopA

*Af*CopA (2 μM) was incubated with increasing concentrations of SDS at 25 °C in mixed micelles composed of C_12_E_10_ and asolectin at a 1:200:200 molar ratio (protein:detergent:lipid). The incubation medium contained 25 mM Tris-HCl pH 7.0, 10 mM NaCl, and 1 mM DTT. Following each SDS addition, samples were incubated for 5 min at 25 °C prior to fluorescence measurements, and fluorescence intensities were corrected for the dilution introduced by the SDS stock solutions, which never exceeded 15% of the initial sample volume. The fluorescence signal remained unchanged between 5 and 30 min after mixing, indicating that equilibrium was reached within the first 5 min.

### 3.4. ATPase Activity

ATPase activity was determined by measuring the initial rate of inorganic phosphate (Pi) release from ATP at 70 °C. The reaction mixture contained 50 mM Tris-HCl (pH 6.1 at 70 °C), 3 mM MgCl_2_, 3 mM ATP, 20 mM cysteine, 130 μM asolectin, 80 μM C_12_E_10_, 800 mM NaCl, 100 μM CuSO_4_, 2.5 mM DTT, and 0.07 μM purified enzyme. The amount of Pi released was quantified using a modified Malachite Green procedure [66,67,68].

### 3.5. Composition of the Micellar Phase

The mole fraction of SDS in the micellar phase was determined using an iterative approach [59] based on the thermodynamic model of mixed micelles described by Tanford [69]. Briefly, the critical micelle concentrations (CMC) of SDS and C_12_E_10_ were experimentally determined in the assay buffer using Isothermal Titration Calorimetry [70]. Asolectin was assumed to be entirely partitioned in the micellar phase due to the extremely low critical micelle concentrations of phospholipids [71]. For each set of total amphiphile concentrations, a system of four equations describing mass balance and micelle-water partition equilibria was solved iteratively. The free concentrations of SDS and C_12_E_10_ in the aqueous phase were adjusted by nonlinear least-squares regression using the Gauss–Newton optimization algorithm [72], allowing calculation of their corresponding mole fractions in the micellar phase. The validity of this approach for the micellar system used in this work was previously demonstrated by showing that the effect of SDS on the intrinsic fluorescence of *Af*CopA at different initial amphiphile concentrations collapsed onto a single behavior when expressed as a function of the mole fraction of SDS in the micellar phase [28].

### 3.6. Steady-State Fluorescence

Fluorescence spectra were collected using a Jasco FP-6500 spectrofluorometer (Jasco, Hachioji, Japan). Tryptophan residues were selectively excited at 295 nm, and emission spectra were collected under different experimental conditions. Spectra were corrected by subtracting the corresponding background signal obtained from protein-free samples prepared in the same incubation medium. The integrated fluorescence intensity was then calculated from the corrected spectra following standard procedures [73]. For thermodynamic analysis, fluorescence intensities were normalized prior to fitting.

(19) $$\mathrm{normalized}\;\mathrm{intensity}=\frac{\left(F-F_{\min}\right)}{\left(F_{\max}-F_{\min}\right)}$$

Fluorescence quantum yields were determined at 25 °C using a relative method [74] with N-acetyltryptophanamide (NATA; *ϕ* = 0.14 [75]) as a reference standard. Quantum yields were calculated from the ratio of integrated fluorescence emission to absorbance at the excitation wavelength, as described before [73]:

(20) $$\phi_{sample}=\phi_{reference}\left(\frac{F_{sample}}{F_{reference}}\right)\cdot \left(\frac{1-10^{-Abs(reference)}}{1-10^{-Abs(sample)}}\right)\cdot {\left(\frac{n_{sample}}{n_{reference}}\right)}^{2}$$

where *Abs* is the absorbance, *F* is the total emission intensity and n is the refractive index.

For the intermediate species, the fluorescence spectra are taken as those of the corresponding vertices in the phasor plot. If a given vertex is not well defined, a matrix decomposition method as described in a previous publication [67] should be used.

### 3.7. Fluorescence Intensity Based Thermodynamic Analysis of SDS-Induced Denaturation

ΔG°_w_ and *m* values were calculated from fluorescence intensity denaturation curves, as described by Eftink [13]. For a two-state model, the corresponding equation is [76]:

(21) $$Signal_{T}=\frac{Signal_{N}+m_{N}\cdot [D]+(Signal_{D}+m_{D}\cdot [D])\cdot e^{-\;\frac{\Delta G_{w,ND}^{\circ }-m_{ND}\cdot [D]}{R\cdot T}}}{1+e^{-\;\frac{\Delta G_{w,ND}^{\circ }-m_{ND}\cdot [D]}{R\cdot T}}}$$

and for the three-state model [77]:

(22) $$\begin{array}{l} Signal_{T}=\frac{\left(Signal_{N}+m_{N}\cdot [D])+K_{NI}\cdot (Signal_{I}+m_{I}\cdot [D])+K_{NI}\cdot K_{ID}\cdot (Signal_{D}+m_{D}\cdot [D]\right)}{1+K_{NI}+K_{NI}\cdot K_{ID}}\\ \mathrm{with}\;K_{NI}=e^{-\;\frac{\Delta G_{w,NI}^{\circ }-m_{NI}\cdot [D]}{R\cdot T}}\;\mathrm{and}\;K_{ID}=e^{-\;\frac{\Delta G_{w,ID}^{\circ }-m_{ID}\cdot [D]}{R\cdot T}} \end{array}$$

where *Signal*_T_ is the signal value corresponding to a given sample, and *Signal*_N_, *Signal*_I_ and *Signal*_D_ are the intrinsic values corresponding to the native, intermediate or denatured states of the protein, respectively, and [D] is the concentration of denaturant (SDS or urea).

### 3.8. Data Analysis

Model parameters were estimated by nonlinear least-squares regression using the Gauss–Newton optimization algorithm [72,78]. For the fitting procedure, the dependent variable was assumed to exhibit constant variance, while uncertainties in the independent variable were considered negligible. Model selection was based on the corrected Akaike Information Criterion (AICc), with preference given to the model yielding the lowest AICc value [79,80]. Joint confidence regions for the regression parameters were calculated as described previously [81].

## 4. Concluding Remarks

In this work, we extended the application of fluorescence spectral phasors from qualitative spectral analysis to the quantitative determination of protein stability parameters.

The linearity of the phasor transformation makes it a powerful framework for describing protein unfolding equilibria. However, these linear relationships are defined in terms of the fraction of detected photons contributed by each fluorescent species, which does not necessarily correspond to molecular populations. By deriving the relationship between photon fractions and molecular fractions and incorporating it into the thermodynamic formalism, we established a rigorous framework for determining protein stability parameters from phasor data.

The performance of the method was first evaluated using simulated fluorescence spectra generated from two- and three-state unfolding models. In all cases, the proposed formalism correctly identified the underlying unfolding mechanism and accurately recovered the values of the thermodynamic parameters used to generate the simulated datasets.

We then applied the method to two experimental systems monitored through tryptophan fluorescence. The first was the urea-induced unfolding of a β-barrel outer membrane protein, in which the tryptophan residues specifically report on the transmembrane domain. For this protein, both conventional intensity-based analysis and spectral phasor analysis yielded a two-state unfolding mechanism and identical thermodynamic parameters once the relationship between photon fractions and molecular populations was taken into account.

The second system was the SDS-induced denaturation of an α-helical membrane protein, where the tryptophan residues report on both the transmembrane domain and the cytosolic catalytic unit. In this case, spectral phasor analysis revealed two unfolding intermediates, whereas conventional fluorescence analysis detected only the intermediate closest to the native state. The clear separation of these intermediates in phasor space enabled the determination of the thermodynamic parameters associated with each of the three unfolding transitions.

In conclusion, the combination of the linear properties of fluorescence phasors with the quantitative relationship between emitted photons and molecular populations establishes spectral phasor analysis as a robust tool for quantifying protein stability. Beyond accurately determining unfolding free energies and their dependence on denaturant concentration, the method provides enhanced sensitivity for detecting intermediate states that may remain hidden using conventional fluorescence analyses, thereby offering a more complete description of complex protein folding landscapes.

## Figures and Tables

**Table 1:** Best-fit parameter values for urea-induced unfolding of W_0_-*Ec*OmpA.

Parameter	Values Reported by Hong et al. [47]	Equation (21) Fitted to Total Intensity Data	Equation (16) Fitted to Relative Distance Data
ΔG°*_w,ND_* (kJ·mol^−1^)	10.3 ± 0.8	10.3 ± 0.7	10.5 ± 0.7
*m* (kJ·mol^−1^·M^−1^)	5.4 ± 0.4	5.4 ± 0.6	5.7 ± 0.3

## Data Availability

Two Python (version 3.11.5) scripts are available on GitHub (https://github.com/alvaroreco/Spectral-Phasor-Quantitative-Analysis, accessed on 14 October 2026). The first converts experimental fluorescence spectra into spectral phasor coordinates and visualizes them in a phasor plot. The second calculates relative distances in phasor space and estimates the fraction of molecules along linear phasor trajectories.

## Supplementary Materials

The following supporting information can be downloaded at: https://www.mdpi.com/article/10.3390/ijms27209233/s1. Reference [82] is cited in the Supplementary Material.

## Abbreviations

SDS	Sodium dodecylsulfate
f*_i_*	Fraction of molecules corresponding to the species *i*
pf*_i_*	Fraction of photons arriving to the detector from the species *i*
*ϕ* *_i_*	Quantum yield of the species *i*
*rd*	Relative distance in the phasor plot
*G_i_*	Real component of the phasor transformation
*S_i_*	Imaginary component of the phasor transformation
*F(λ)_i_*	Fluorescence intensity at wavelength λ corresponding to the species *i*
*Ec*OmpA	Outer membrane protein A from *E. coli*
*Af*CopA	Cu(I) transport ATPase from *A. fulgidus*

## Appendix A. On the Relationship Between the Mole Fraction of Molecules in a Mixture of Fluorophores and the Fraction of Photons Emitted from Each Species

In a mixture of fluorophores, the fraction of photons reaching the detector from each species is defined as:

(A1) $${\mathrm{pf}}_{i}\;=\;\frac{\mathrm{number}\;\mathrm{of}\;\mathrm{photons}\;\mathrm{from}\;\mathrm{the}\;\mathrm{species}\;i}{\mathrm{total}\;\mathrm{number}\;\mathrm{of}\;\mathrm{photons}\;\mathrm{reaching}\;\mathrm{the}\;\mathrm{detector}}$$

Considering the quantum yield of each species (*ϕ**_i_*), this fraction can be expressed in terms of the number of photons absorbed by each species (*n_abs,i_*),

(A2) $${\mathrm{pf}}_{i}=\frac{n_{abs,i}\cdot \phi_{i}}{\sum_{i}\;n_{abs,i}\cdot \phi_{i}}$$

and the number of absorbed photons is related to the number of incident photons (*n_inc_*) according to Beer and Lambert’s law,

(A3) $$n_{abs,i}=n_{inc}\cdot \left(1-10^{-\varepsilon_{i}\cdot c_{i}\cdot l}\right)$$

where *ε_i_* is the molar absorption coefficient of the species *i*, *c_i_* the molar concentration of each species, and *l* the optical path length.

Expanding the exponential term in Equation (A3) into a first-order Taylor series:

(A4) $$n_{abs,i}≅n_{inc}\cdot \left(1-1+\ln10\cdot \varepsilon_{i}\cdot c_{i}\cdot l\right)$$

Figure S9 shows that this approximation produces a negligible error in *n_abs,i_* for very low sample absorbance values. As a practical rule, it is valid for samples with absorbances below 0.05 at the excitation wavelength for which the relative error is about 0.6%, and the internal filter effect is also negligible [73].

Replacing Equation (A4) in (A2) and simplifying the terms that appear in the numerator and denominator, and dividing the numerator and denominator by the total molar concentration,

(A5) $${\mathrm{pf}}_{i}=\frac{n_{inc}\cdot \ln10\cdot \varepsilon_{i}\cdot c_{i}\cdot l\cdot \phi_{i}}{\sum_{i}n_{inc}\cdot \ln10\cdot \varepsilon_{i}\cdot c_{i}\cdot l\cdot \phi_{i}}=\frac{\varepsilon_{i}\cdot c_{i}\cdot \phi_{i}}{\sum_{i}\varepsilon_{i}\cdot c_{i}\cdot \phi_{i}}=\frac{\varepsilon_{i}\cdot f_{i}\cdot \phi_{i}}{\sum_{i}\varepsilon_{i}\cdot f_{i}\cdot \phi_{i}}$$

where f*_i_* is the fraction of molecules corresponding to the species *i* (c_i_/∑c_i_). It should be noted that, for both the photon’s fraction and the molecule’s fraction, the conservation principle is fulfilled:

(A6) $$\sum_{i}{\mathrm{pf}}_{i}\;=\;1$$

(A7) $$\sum_{i}f_{i}=1$$

For a two-state process, only two species emit photons; therefore,

(A8) $${\mathrm{pf}}_{1}=\frac{\varepsilon_{1}\cdot f_{1}\cdot \phi_{1}}{\varepsilon_{1}\cdot f_{1}\cdot \phi_{1}+\varepsilon_{2}\cdot f_{2}\cdot \phi_{2}}$$

If the molar absorption coefficients of both states are equal, Equation (A8) reduces to

(A9) $${\mathrm{pf}}_{1}=\frac{f_{1}\cdot \phi_{1}}{f_{1}\cdot \phi_{1}+\left({1\;-\;f}_{1}\right)\cdot \phi_{2}}$$

and solving for f_1_

(A10) $$f_{1}=\frac{{\mathrm{pf}}_{1}\cdot \phi_{2}}{\phi_{1}+{\mathrm{pf}}_{1}\cdot (\phi_{2}-\phi_{1})}$$

## References

[1] Tanford C. (1970). Protein Denaturation: Part C. Theoretical Models for The Mechanism of Denaturation. Adv. Protein Chem..

[2] Privalov P.L. (1979). Stability of Proteins Small Globular Proteins. Adv. Protein Chem..

[3] Roman E.A., González Flecha F.L. (2014). Kinetics and Thermodynamics of Membrane Protein Folding. Biomolecules.

[4] Schellman J.A. (2003). Protein Stability in Mixed Solvents: A Balance of Contact Interaction and Excluded Volume. Biophys. J..

[5] Myers J.K., Pace C.N., Scholtz J.M. (1995). Denaturant *m* Values and Heat Capacity Changes: Relation to Changes in Accessible Surface Areas of Protein Unfolding. Protein Sci..

[6] Courtenay E.S., Capp M.W., Record M.T. (2001). Thermodynamics of Interactions of Urea and Guanidinium Salts with Protein Surface: Relationship between Solute Effects on Protein Processes and Changes in Water-Accessible Surface Area. Protein Sci..

[7] Courtenay E.S., Capp M.W., Saecker R.M., Record M.T. (2000). Thermodynamic Analysis of Interactions between Denaturants and Protein Surface Exposed on Unfolding: Interpretation of Urea and Guanidinium Chloride *m*-Values and Their Correlation with Changes in Accessible Surface Area (ASA) Using Preferential Interaction Coefficients and the Local-Bulk Domain Model. Proteins.

[8] Scholtz J.M., Grimsley G.R., Pace C.N. (2009). Solvent Denaturation of Proteins and Interpretations of the *m* Value. Methods Enzymol..

[9] Jackson S.E., Fersht A.R. (1991). Folding of Chymotrypsin Inhibitor 2. 1. Evidence for a Two-State Transition. Biochemistry.

[10] Kuwajima K. (2020). The Molten Globule, and Two-State vs. Non-Two-State Folding of Globular Proteins. Biomolecules.

[11] Jackson S.E. (1998). How Do Small Single-Domain Proteins Fold?. Structure.

[12] Barrick D. (2009). What Have We Learned from the Studies of Two-State Folders, and What Are the Unanswered Questions about Two-State Protein Folding?. Phys. Biol..

[13] Eftink M.R. (1994). The Use of Fluorescence Methods to Monitor Unfolding Transitions in Proteins. Biophys. J..

[14] Royer C.A. (2006). Probing Protein Folding and Conformational Transitions with Fluorescence. Chem. Rev..

[15] Ladokhin A.S., Jayasinghe S., White S.H. (2000). How to Measure and Analyze Tryptophan Fluorescence in Membranes Properly, and Why Bother?. Anal. Biochem..

[16] Moon C.P., Fleming K.G. (2011). Using Tryptophan Fluorescence to Measure the Stability of Membrane Proteins Folded in Liposomes. Methods Enzymol..

[17] Ranjit S., Malacrida L., Jameson D.M., Gratton E. (2018). Fit-Free Analysis of Fluorescence Lifetime Imaging Data Using the Phasor Approach. Nat. Protoc..

[18] Malacrida L., Ranjit S., Jameson D.M., Gratton E. (2021). The Phasor Plot: A Universal Circle to Advance Fluorescence Lifetime Analysis and Interpretation. Annu. Rev. Biophys..

[19] Malacrida L. (2023). Phasor Plots and the Future of Spectral and Lifetime Imaging. Nat. Methods.

[20] Jameson D.M., Gratton E., Hall R.D. (1984). The Measurement and Analysis of Heterogeneous Emissions by Multifrequency Phase and Modulation Fluorometry. Appl. Spectrosc. Rev..

[21] Reinhart G.D., Marzola P., Jameson D.M., Gratton E. (1991). A Method for On-Line Background Subtraction in Frequency Domain Fluorometry. J. Fluoresc..

[22] Digman M.A., Caiolfa V.R., Zamai M., Gratton E. (2008). The Phasor Approach to Fluorescence Lifetime Imaging Analysis. Biophys. J..

[23] Weber G. (1981). Resolution of the Fluorescence Lifetimes in a Heterogeneous System by Phase and Modulation Measurements. J. Phys. Chem..

[24] Fereidouni F., Bader A.N., Gerritsen H.C. (2012). Spectral Phasor Analysis Allows Rapid and Reliable Unmixing of Fluorescence Microscopy Spectral Images. Opt. Express.

[25] Recoulat Angelini A.A., Malacrida L., González Flecha F.L. (2025). Fluorescence Phasor Analysis: Basic Principles and Biophysical Applications. Biophys. Rev..

[26] Bader A.N., Visser N.V., van Amerongen H., Visser A.J.W.G. (2014). Phasor Approaches Simplify the Analysis of Tryptophan Fluorescence Data in Protein Denaturation Studies. Methods Appl. Fluoresc..

[27] Cruz Rodríguez L., Foressi N.N., Celej M.S. (2025). Tracking Protein Transitions through Fluorescence Spectral Phasor Analysis with ACDAN. Biophys. Rep..

[28] Recoulat Angelini A.A., Roman E.A., González Flecha F.L. (2024). The Structural Stability of Membrane Proteins Revisited: Combined Thermodynamic and Spectral Phasor Analysis of SDS-Induced Denaturation of a Thermophilic Cu(I)-Transport ATPase. J. Mol. Biol..

[29] Cattoni D.I., González Flecha F.L., Argüello J.M. (2008). Thermal Stability of CopA, a Polytopic Membrane Protein from the Hyperthermophile *Archaeoglobus fulgidus*. Arch. Biochem. Biophys..

[30] Roman E.A., Argüello J.M., González Flecha F.L. (2010). Reversible Unfolding of a Thermophilic Membrane Protein in Phospholipid/Detergent Mixed Micelles. J. Mol. Biol..

[31] Torrado B., Malacrida L., Ranjit S. (2022). Linear Combination Properties of the Phasor Space in Fluorescence Imaging. Sensors.

[32] Villar S.F., Dalla-Rizza J., Möller M.N., Ferrer-Sueta G., Malacrida L., Jameson D.M., Denicola A. (2022). Fluorescence Lifetime Phasor Analysis of the Decamer-Dimer Equilibrium of Human Peroxiredoxin 1. Int. J. Mol. Sci..

[33] Burstein E.A., Abornev S.M., Reshetnyak Y.K. (2001). Decomposition of Protein Tryptophan Fluorescence Spectra into Log-Normal Components. I. Decomposition Algorithms. Biophys. J..

[34] Ye C., Wang Z., Lu W., Wei Y. (2014). Unfolding Study of a Trimeric Membrane Protein AcrB. Protein Sci..

[35] Krainer G., Hartmann A., Bogatyr V., Nielsen J., Schlierf M., Otzen D.E. (2020). SDS-Induced Multi-Stage Unfolding of a Small Globular Protein through Different Denatured States Revealed by Single-Molecule Fluorescence. Chem. Sci..

[36] Soulages J.L. (1998). Chemical Denaturation: Potential Impact of Undetected Intermediates in the Free Energy of Unfolding and *m*-Values Obtained from a Two-State Assumption. Biophys. J..

[37] Mahalakshmi R. (2026). Decoding Outer Membrane β-Barrels: From Structural Curiosity to Engineered Nanotherapeutics. Chem. Rev..

[38] Wimley W.C. (2002). Toward Genomic Identification of β-Barrel Membrane Proteins: Composition and Architecture of Known Structures. Protein Sci..

[39] Noinaj N., Rollauer S.E., Buchanan S.K. (2015). The β-Barrel Membrane Protein Insertase Machinery from Gram-Negative Bacteria. Curr. Opin. Struct. Biol..

[40] Kleinschmidt J.H. (2015). Folding of β-Barrel Membrane Proteins in Lipid Bilayers—Unassisted and Assisted Folding and Insertion. Biochim. Biophys. Acta.

[41] Schiffrin B., Brockwell D.J., Radford S.E. (2017). Outer Membrane Protein Folding from an Energy Landscape Perspective. BMC Biol..

[42] Wang X., Bernstein H.D. (2022). The *Escherichia coli* Outer Membrane Protein OmpA Acquires Secondary Structure Prior to Its Integration into the Membrane. J. Biol. Chem..

[43] Pautsch A., Schulz G.E. (2000). High-Resolution Structure of the OmpA Membrane Domain. J. Mol. Biol..

[44] Ishida H., Garcia-Herrero A., Vogel H.J. (2014). The Periplasmic Domain of *Escherichia coli* Outer Membrane Protein A Can Undergo a Localized Temperature Dependent Structural Transition. Biochim. Biophys. Acta.

[45] White S., Heijne G.v., Engelman D. (2021). Cell Boundaries: How Membranes and Their Proteins Work.

[46] Fleming K.G. (2014). Energetics of Membrane Protein Folding. Annu. Rev. Biophys..

[47] Hong H., Rinehart D., Tamm L.K. (2013). Membrane Depth-Dependent Energetic Contribution of the Tryptophan Side Chain to the Stability of Integral Membrane Proteins. Biochemistry.

[48] Ubarretxena-Belandia I., Engelman D.M. (2001). Helical Membrane Proteins: Diversity of Functions in the Context of Simple Architecture. Curr. Opin. Struct. Biol..

[49] Krogh A., Larsson B., von Heijne G., Sonnhammer E.L.L. (2001). Predicting Transmembrane Protein Topology with a Hidden Markov Model: Application to Complete Genomes. J. Mol. Biol..

[50] Sánchez Lorente J., Sokolov A.V., Ferguson G., Schiöth H.B., Hauser A.S., Gloriam D.E. (2025). GPCR Drug Discovery: New Agents, Targets and Indications. Nat. Rev. Drug Discov..

[51] Mandal A.K., Cheung W.D., Argüello J.M. (2002). Characterization of a Thermophilic P-Type Ag^+^/Cu^+^-ATPase from the Extremophile *Archaeoglobus fulgidus*. J. Biol. Chem..

[52] Argüello J.M., Eren E., González-Guerrero M. (2007). The Structure and Function of Heavy Metal Transport P1B-ATPases. Biometals.

[53] González-Guerrero M., Argüello J.M. (2008). Mechanism of Cu^+^-Transporting ATPases: Soluble Cu^+^ Chaperones Directly Transfer Cu^+^ to Transmembrane Transport Sites. Proc. Natl. Acad. Sci. USA.

[54] González-Guerrero M., Eren E., Rawat S., Stemmler T.L., Argüello J.M. (2008). Structure of the Two Transmembrane Cu^+^ Transport Sites of the Cu^+^-ATPases. J. Biol. Chem..

[55] Salustros N., Grønberg C., Abeyrathna N.S., Lyu P., Orädd F., Wang K., Andersson M., Meloni G., Gourdon P. (2022). Structural Basis of Ion Uptake in Copper-Transporting P1B-Type ATPases. Nat. Commun..

[56] Andersson M., Mattle D., Sitsel O., Klymchuk T., Nielsen A.M., Møller L.B., White S.H., Nissen P., Gourdon P. (2014). Copper-Transporting P-Type ATPases Use a Unique Ion-Release Pathway. Nat. Struct. Mol. Biol..

[57] Lyu P., Longhin E., Sabzian-Molaei F., Montalvillo Ortega F., Li P., Wang K., Duelli A., Al-Jubair T., Ahmed Hotiana H., Ramos Becares E. (2026). Structural and Functional Insights into a Zn^2+^-Transporting P-Type ATPase. Nat. Commun..

[58] Recoulat Angelini A.A., Placenti M.A., Melian N.A., Sabeckis M.L., Burgardt N.I., González Lebrero R.M., Roman E.A., González Flecha F.L. (2021). Cu(I)-Transport ATPases. Molecular Architecture, Catalysis and Adaptation to Extreme Environments. Adv. Med. Biol..

[59] Recoulat Angelini A.A., Incicco J.J., Melian N.A., González-Flecha F.L. (2023). Susceptibility of Cu(I) Transport ATPases to Sodium Dodecyl Sulfate. Relevance of the Composition of the Micellar Phase. Arch. Biochem. Biophys..

[60] Chang Y.-C., Bowie J.U. (2014). Measuring Membrane Protein Stability under Native Conditions. Proc. Natl. Acad. Sci. USA.

[61] Bredeston L.M., González Flecha F.L. (2016). The Promiscuous Phosphomonoestearase Activity of *Archaeoglobus fulgidus* CopA, a Thermophilic Cu^+^ Transport ATPase. Biochim. Biophys. Acta.

[62] Liu X., Sitsel O., Wang K., Gourdon P., Bublitz M. (2016). Overproduction of PIB-Type ATPases. P-Type ATPases: Methods and Protocols.

[63] Schägger H. (2006). Tricine–SDS-PAGE. Nat. Protoc..

[64] Gill S.C., von Hippel P.H. (1989). Calculation of Protein Extinction Coefficients from Amino Acid Sequence Data. Anal. Biochem..

[65] Pilkington T.J., Bignucolo M.W., Ainsworth J.P., Siemann S. (2023). An Excel Fitting Routine for Correcting Protein Absorption Spectra for Scatter. Anal. Biochem..

[66] Martínez Gache S.A., Recoulat Angelini A.A., Sabeckis M.L., González Flecha F.L. (2020). Improving the Stability of the Malachite Green Method for the Determination of Phosphate Using Pluronic F68. Anal. Biochem..

[67] Recoulat Angelini A.A., Martínez Gache S.A., Sabeckis M.L., Melian N.A., González Flecha F.L. (2022). On the Role of Citrate in 12-Molybdophosphoric-Acid Methods for Quantification of Phosphate in the Presence of ATP. New J. Chem..

[68] Recoulat Angelini A.A., Gómez G.E., Delfino J.M., González Flecha F.L. (2026). A Sensitive Smartphone-Based Method for Measuring Phosphate in Biological Samples and ATPase Activities. Talanta.

[69] Tanford C. (1980). The Hydrophobic Effect: Formation of Micelles and Biological Membranes.

[70] Tso S.-C., Mahler F., Höring J., Keller S., Brautigam C.A. (2020). Fast and Robust Quantification of Detergent Micellization Thermodynamics from Isothermal Titration Calorimetry. Anal. Chem..

[71] Scollo F., Tempra C., Lolicato F., Sciacca M.F.M., Raudino A., Milardi D., La Rosa C. (2018). Phospholipids Critical Micellar Concentrations Trigger Different Mechanisms of Intrinsically Disordered Proteins Interaction with Model Membranes. J. Phys. Chem. Lett..

[72] Seber G.A.F., Wild C.J. (2005). Nonlinear Regression.

[73] Jameson D.M. (2025). Introduction to Fluorescence.

[74] Parker C.A., Rees W.T. (1960). Correction of Fluorescence Spectra and Measurement of Fluorescence Quantum Efficiency. Analyst.

[75] Szabo A.G., Rayner D.M. (1980). Fluorescence Decay of Tryptophan Conformers in Aqueous Solution. J. Am. Chem. Soc..

[76] Santoro M.M., Bolen D.W. (1988). Unfolding Free Energy Changes Determined by the Linear Extrapolation Method. 1. Unfolding of Phenylmethanesulfonyl Alpha-Chymotrypsin Using Different Denaturants. Biochemistry.

[77] Sancho J. (2013). The Stability of 2-State, 3-State and More-State Proteins from Simple Spectroscopic Techniques... plus the Structure of the Equilibrium Intermediates at the Same Time. Arch. Biochem. Biophys..

[78] Kemmer G., Keller S. (2010). Nonlinear Least-Squares Data Fitting in Excel Spreadsheets. Nat. Protoc..

[79] Akaike H. (1978). A Bayesian Analysis of the Minimum AIC Procedure. Ann. Inst. Stat. Math..

[80] Burnham K.P., Anderson D.R. (2002). Advanced Issues and Deeper Insights. Model Selection and Multimodel Inference: A Practical Information-Theoretic Approach.

[81] Box G.E.P., Hunter J.S., Hunter W.G. (2005). Statistics for Experimenters: Design, Innovation, and Discovery.

[82] Jo S., Kim T., Iyer V.G., Im W. (2008). CHARMM-GUI: A Web-Based Graphical User Interface for CHARMM. J. Comput. Chem..

