# Supplementary material for: Linear Combination Properties of Spectral Phasors Allow Thermodynamic Analysis of Reversible Denaturation of Multidomain and Membrane Proteins
Source: Int J Mol Sci. 2026 Oct 17;27(20):9233. doi: 10.3390/ijms27209233 (PMC13704976; doi:10.3390/ijms27209233)
Supplement: Supplementary file 1 [file ijms-27-09233-s001.zip › ijms-4576870-supplementary.pdf]

Linear Combination Properties of Spectral Phasors Allow Thermodynamic Analysis of Reversible Denaturation of Multidomain and Membrane Proteins

Alvaro A. Recoulat Angelini, Leonel Malacrida and F. Luis González Flecha

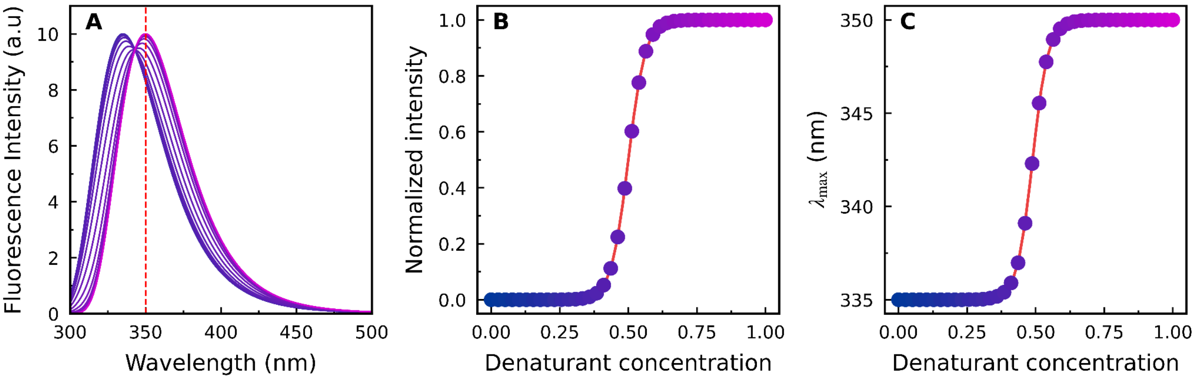

Figure S1. Traditional thermodynamic analysis of a two-state denaturation process exhibiting a fluorescence red shift without changes in the quantum yield. (**A**) Fluorescence spectra were simulated as described in Figure 1. (**B**) The fluorescence intensity at 350 nm (dashed red line in panel A) was normalized using Equation (19) and plotted as a function of denaturant concentration (expressed as relative to the maximal concentration). Equation (21) was fitted to these data and the best-fit parameter values were $\Delta G°_{w,ND}$ = 40.0 ± 10^{-6} kJ/mol and m_{ND} = 80.0 ± 10^{-6} kJ/mol. (**C**) The wavelength of the maximum intensity (λ_{max}) was plotted as a function of denaturant concentration and Equation (21) was fitted to these data with the best-fit parameter values were $\Delta G°_{w,ND}$ = 42.7 ± 0.1 kJ/mol and m_{ND} = 87.3 ± 0.3 kJ/mol. It can be observed that thermodynamic analysis of fluorescence intensity data allows for the recovery of the original parameter values used in the simulations, whereas the same analysis applied to the wavelength of maximal intensity data leads to an incorrect parameter estimation despite the good fit.

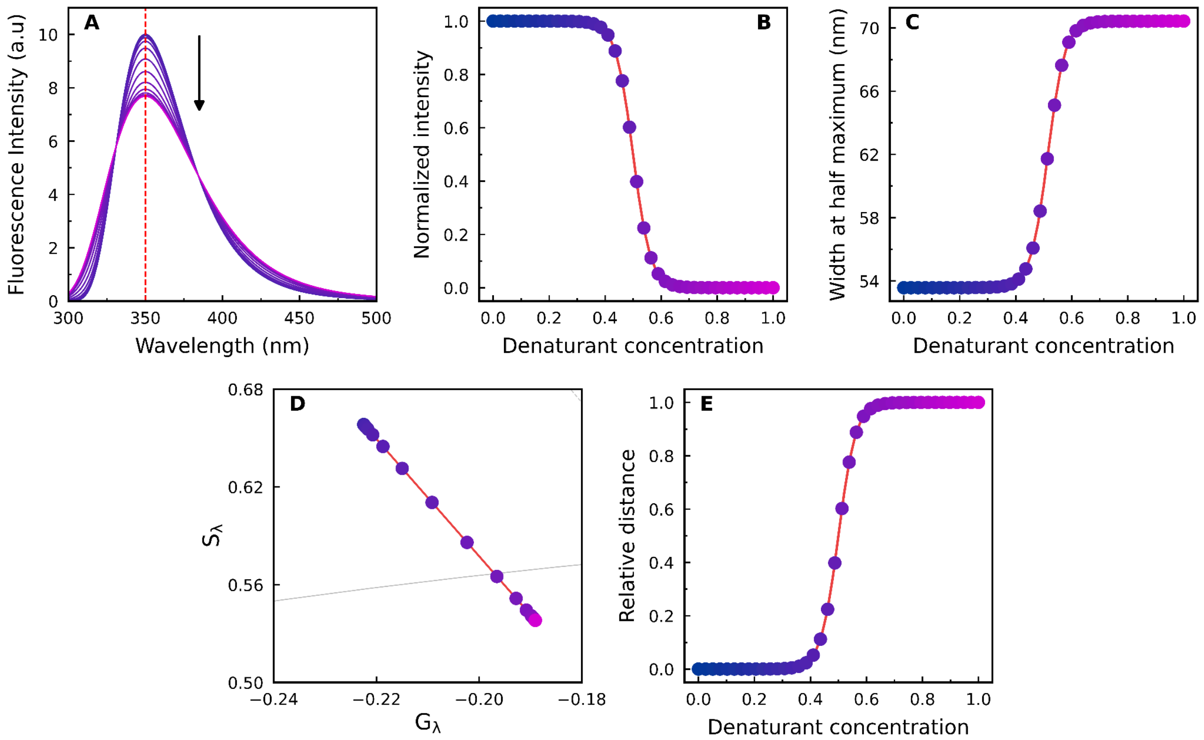

Figure S2. Simulation of a two-state denaturation process exhibiting spectral broadening without changes in the quantum yield. (**A**) Fluorescence spectra of the native (blue) and denatured (purple) states were simulated using a log-normal function [33]. Both spectra were simulated with the same emission maxima (350 nm) but with different spectral width at half maximum. A denaturation process was modeled using Equations (2) and (3) with $\Delta G°_{w,ND}$ = 40 kJ/mol and m_{ND} = 80 kJ/mol, yielding the fractions of native (f_N) and denatured (f_D) species as a function of denaturant concentration. With this data, the spectra along the denaturation process were constructed as a linear combination of the native and denatured spectra (Equation (17)). The arrow indicates increasing denaturant concentration. (**B**) The fluorescence intensity at 350 nm (dashed red line in panel A) was normalized using Equation (19) and plotted as a function of denaturant concentration (expressed as relative to the maximal concentration). Equation (21) was fitted to these data, and the best-fit parameter values were $\Delta G°_{w,ND}$ = 40 ± 10^{-6} kJ/mol and m_{ND} = 80.0 ± 10^{-6} kJ/mol. (**C**) The full width at half of the maximum intensity was plotted as a function of denaturant concentration. Equation (21) was fitted to these data, and the best-fit parameter values were $\Delta G°_{w,ND}$ = 41.78 ± 0.03 kJ/mol and m_{ND} = 81.14 ± 0.05 kJ/mol. (**D**) Phasor components of each spectrum shown in panel A were calculated using Equations (6). (**E**) The relative distances in the phasor plot ($d_{i,N}/d_{D,N}$) were calculated using Equation (18) and plotted as a function of the denaturant concentration. Equation (16), corresponding to a two-state model, was fitted to these data. The best fit parameter values were $\Delta G°_{w,ND}$ = 40.0 ± 10^{-6} kJ/mol and m_{ND} = 80.0 ± 10^{-6}kJ/mol. As can be observed, both the thermodynamic analysis of fluorescence intensity and the quantitative phasor approach allow recovering the original values used in the simulation. On the contrary, thermodynamic analysis of the spectral width at half maximum data leads to an incorrect parameter estimation despite the good fit.

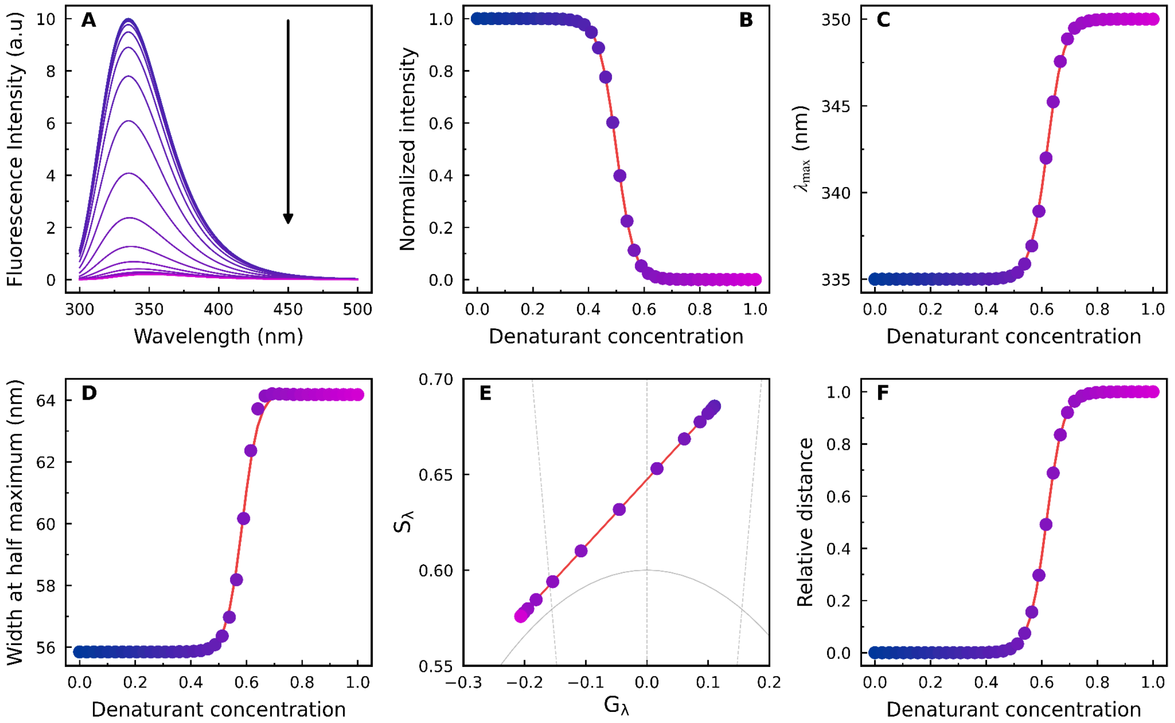

Figure S3. Simulation of a two-state denaturation process exhibiting changes in the fluorescence quantum yield, emission maximum and spectral width at half maximum. (**A**) Fluorescence spectra of the native (blue) and denatured (purple) states were simulated using a log-normal function [33]. Both spectra were simulated with different spectral width at half maximum, shifted emission maxima (native at 335 nm and denatured at 350 nm), and different quantum yields. A denaturation process was modeled using Equations (2) and (3) with $\Delta G°_{w,ND}$ = 40 kJ/mol and m_{ND} = 80 kJ/mol, yielding the fractions of native (f_N) and denatured (f_D) species as a function of denaturant concentration. With this data, the spectra along the denaturation process were constructed as a linear combination of the native and denatured spectra (Equation (17)). The arrow indicates increasing denaturant concentration. (**B**) The total fluorescence intensity was calculated, normalized with Equation (19) and plotted as a function of denaturant concentration (expressed as relative to the maximal concentration). Equation (21) was fitted to these data, and the best-fit parameter values were $\Delta G°_{w,ND}$ = 40.0 ± 10^{-6} kJ/mol and m_{ND} = 80.0 ± 10^{-6} kJ/mol. (**C**) The wavelength of the maximum intensity (λ_{max}) was plotted as a function of denaturant concentration. Equation (21) was fitted to these data, and the best-fit parameter values were $\Delta G°_{w,ND}$ = 50.0 ± 0.5 kJ/mol and m_{ND} = 80.7 ± 0.8 kJ/mol. (**D**) The full width at half of the maximum intensity was plotted as a function of denaturant concentration. Equation (21) was fitted to these data, and the best-fit parameter values were $\Delta G°_{w,ND}$ = 50.0 ± 0.2 kJ/mol and m_{ND} = 85 ± 3 kJ/mol. (**E**) Phasor components of each spectrum shown in panel A were calculated using Equations (6) (**F**) The relative distances in the phasor plot ($d_{i,N}/d_{D,N}$) were calculated using Equation (18) and plotted as a function of the denaturant concentration. Equation (16), with $\phi_N = \phi_D$, was fitted to these data (purple line). The best fit parameter values were $\Delta G°_{w,ND}$ = 50.0 ± 10^{-6} kJ/mol and m_{ND} = 80.0 ± 10^{-6} kJ/mol. Alternatively, Equation (16) with with $\phi_N / \phi_D = 50$ (derived from the spectra shown in panel A) was also fitted to these data (red line). It is worth noting that both fittings are indistinguishable, but in the last case the best fit parameter values were $\Delta G°_{w,ND}$ = 40.0 ± 10^{-6} kJ/mol and m_{ND} = 80.0 ± 10^{-6} J/mol, thus recovering the original values used in the simulation.

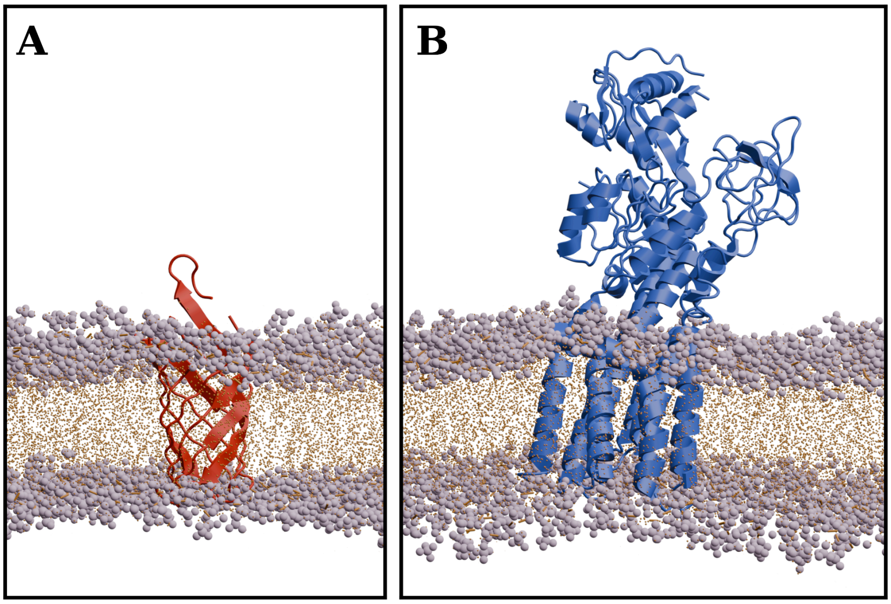

Figure S4. Three-dimensional structures of *Ec*OmpA and *Af*CopA. The atomic coordinates of *Ec*OmpA and *Af*CopA were obtained from the Protein Data Bank (PDB IDs 1QJP [43] and 7R0I [55], respectively) and embedded in a 1,2-dimyristoyl-sn-glycero-3-phosphocholine (DMPC) bilayer using the CHARMM-GUI web-based platform [82]. *Ec*OmpA is shown in red (panel A) and *Af*CopA in blue (panel B). DMPC phospholipids are represented by their hydrophobic tails (yellow) and hydrophilic head groups (violet). The images were rendered using Blender (http://www.blender.org).

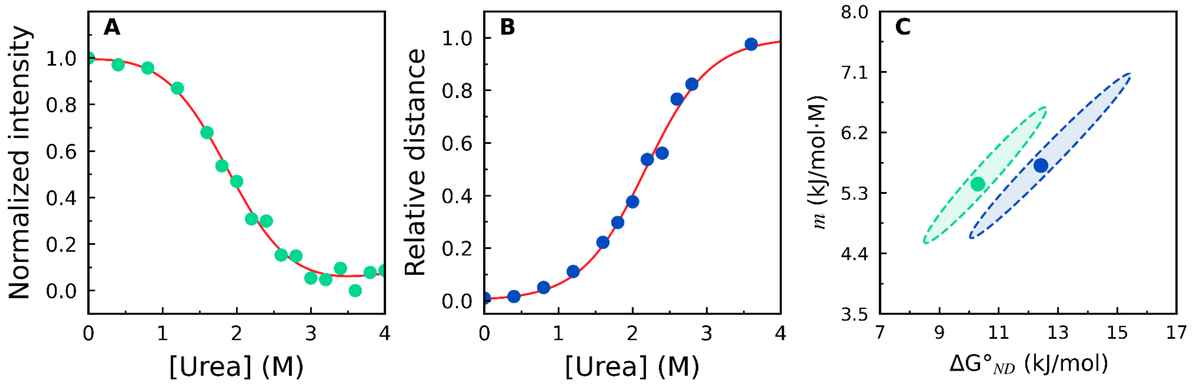

Figure S5. Fitting a two-state model without considering the differences in quantum yields to the urea-induced denaturation data of OmpA. A two-state model was fitted to urea-induced denaturation data of *Ec*OmpA shown in Figure 3 without considering the differences in quantum yields. Equation (21) was fitted to the normalized total intensity (A), and Equation (16) with $\phi_N = \phi_D$ to the relative distances in the phasor plot between the native and unfolded states (B). The points in panel C represent the best-fit values of $\Delta G°_{w,ND}$ (10.3 ± 1.7 kJ·mol^{-1} from total intensity and 12.4 ± 0.7 kJ·mol^{-1} from relative distance) and m_{ND} (5.4 ± 0.6 kJ·mol^{-1} M^{-1} from total intensity and 5.7 ± 0.3 kJ·mol^{-1} M^{-1} from relative distance), while the dotted lines indicate the 99% confidence regions (green for intensity data and blue for relative distances in the phasor plot).

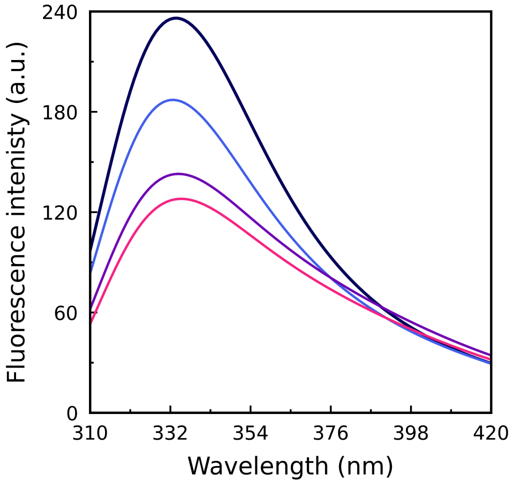

Figure S6. Fluorescence spectra of the four species involved in SDS-induced denaturation of *Af*CopA. *Af*CopA (2 µM) was incubated for 5 min at 25°C in the medium described in Materials and Methods with increasing concentrations of SDS in two independent titrations. Intrinsic fluorescence spectra were recorded between 310 and 420 nm after excitation at 295 nm. Based on phasor analysis (Figure 4C), the selected spectra represent those of the native state (dark blue line), intermediary state 1 (blue line), intermediary state 2 (purple line), and denatured state (pink line), corresponding to mole fractions of SDS of 0, 0.25, 0.84, and 0.95, respectively.

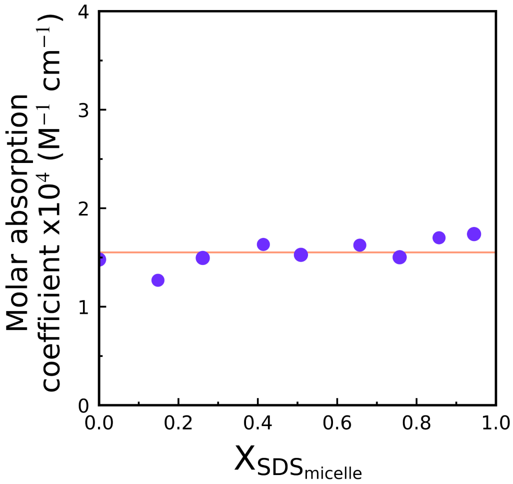

Figure S7. Molar absorption coefficients of *Af*CopA at different SDS mole fractions in the micelle. Aliquots of *Af*CopA (2 µM) were incubated for 5 min at 25 °C in the presence of varying SDS concentrations. Absorbance at 295 nm was measured, and the molar absorption coefficients were calculated and plotted as a function of the mole fraction of SDS in the micelle (see Materials and Methods for details).

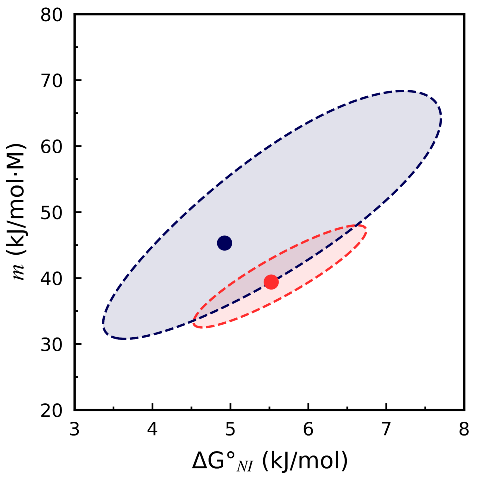

Figure S8. Confidence regions for the stability parameters of the first transition of SDS induced denaturation of *Af*CopA. Thermodynamic analysis of the first transition of SDS induced denaturation of *Af*CopA (Figure 5A,D) was performed on the total intensity data (red), and the first phasor plot trajectory (blue). The points represent the best-fit values of $\Delta G^{\circ}_{w,NI}$ (5.5 ± 0.5 kJ·mol^{-1} from total intensity and 4.9 ± 0.6 kJ·mol^{-1} from relative distance) and m_{NI} (40 ± 3 kJ·mol^{-1} M^{-1} from total intensity and 45 ± 6 kJ·mol^{-1} M^{-1} from relative distance), while the dotted lines indicate the 99% confidence regions.

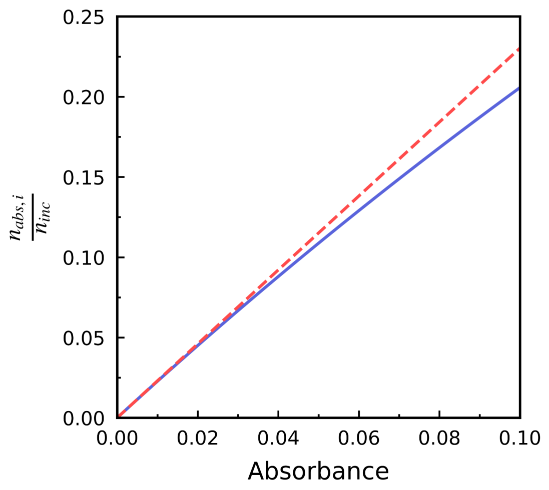

Figure S9. Relation between the absorbance and the relative number of absorbed photons. The number of absorbed photons ($n_{abs,i}$) relative to the number of incident photons (n_{inc}) was calculated using the Beer and Lambert's law (Equation (A3) - solid blue line) and a first-order Taylor series approximation (Equation (A4) - red dotted line).

Table S1. Quantum yields of the four identified species on the SDS-induced denaturation pathway of *Af*CopA

Folding state	Quantum yield
Native (N)	0.18 ± 0.01
Intermediary 1 (I_1)	0.15 ± 0.01
Intermediary 2 (I_2)	0.13 ± 0.01
Denatured (D)	0.12 ± 0.01
